# Impacts of entropy generation in second-grade fuzzy hybrid nanofluids on exponentially permeable stretching/shrinking surface

**DOI:** 10.1038/s41598-023-48142-0

**Published:** 2023-12-13

**Authors:** Rana Muhammad Zulqarnain, Muhammad Nadeem, Imran Siddique, Adeel Mansha, Abdullatif Saleh Ghallab, Mahvish Samar

**Affiliations:** 1 School of Mathematical Sciences, Zhejiang Normal University, Jinhua, 321004 Zhejiang China; 2 Department of Mathematics, University of Management and Technology, Lahore, 54770 Pakistan; 3 Department of Mathematics, University of Sargodha, Sargodha, 40100 Pakistan; 4 Department of Physics, Zhejiang Normal University, Jinhua, 321004 Zhejiang China; 5 Department of Computer Science, University of Science and Technology, P.O. Box: 13064, Sanaa, Yemen

**Keywords:** Mathematics and computing, Physics

## Abstract

The present investigation aims to use entropy analysis to analyze the unsteady Magnetohydrodynamic (MHD) flow in a second-grade fuzzy hybrid $\left( {{\text{Al}}_{{2}} {\text{O}}_{{3}} - {\text{Cu/SA}}} \right)$ nanofluid over an exponentially shrinking/stretching surface. The model for hybridization of the mixture of alumina $\left( {{\text{Al}}_{{2}} {\text{O}}_{{3}} } \right)$ and copper (Cu) nanoparticles in the sodium alginate (SA) base fluid under heat source/sink, nonlinear thermal radiation, and viscous dissipation. The fundamental partial differential equations (PDEs) are simplified using an appropriate similarity conversion to generate the ordinary differential equations (ODEs). The analytical computation occurs in the MATHEMATICA program implementing the homotopy analysis method (HAM). In terms of code validity, our results are preferable to previous findings. The features of several parameters against the velocity, surface friction coefficient, entropy, temperature, and Nusselt number are described through graphs. According to our findings, the rise in the Brinkman and Reynolds numbers enhanced the total entropy of the system. Furthermore, the nanoparticle volume fraction and viscus dissipation magnifies the fluid temperature while retards the flow profile throughout the domain. Fluid velocity declined due to the Lorentz force using magnetic impact applications. The imprecision of nanofluid and hybrid nanofluid volume fractions was modelled as a triangular fuzzy number (TFN) [0%, 1%, 2%] for comparison. The double parametric approach was applied to deal with the fuzziness of the associated fuzzy parameters. The nonlinear ODEs convert into fuzzy differential equations (FDEs) and use HAM for the fuzzy solution. From our observation, the hybrid nanofluid displays the maximum heat transfer compared to nanofluids. This important contribution will support industrial growth, particularly in the processing and manufacturing sectors. The percentage increase in skin friction factor is 18.3 and 15.0 when $\alpha$ and $\beta$ take input in the ranges of 0 ≤ $\alpha$ ≤ 0.8 and 0 ≤ $\beta$ ≤ 1, respectively.

## Introduction

Non-Newtonian materials have attracted the attention of investigators for over a century because of their interdisciplinary characteristics and stimulating texture. Such fluids have been used in various industries, including manufacturing, metallic substances, chemical processing, materials for plastics, and foodstuffs. Glassware exhaling, biologic fluids, skin care products, synthetic fibers, healthcare, food, metallic twirling, shampoos and conditioners, and numerous other sectors use non-Newtonian fluids. These fluids possess a broad range of features and are categorized as dilatant, shear-thickening, shear-thinning fluids, or thixotropic. Rheologists have identified and studied various fluid simulations: Casson, Williamson, Maxwell, third grade, Burgers, micropolar, Oldroyd-B, Sutterby Cross, Jeffrey, and Sisko. But second-grade fluid behaves differently under different conditions, which explains the characteristics of shear-thickening/thinning and Newtonian effects. According to its dynamical properties, second-grade fluid is acclaimed and esteemed among researchers^1–4^.

Stretching a plastic sheet, on the other hand, is not always linear. An exponentially stretched sheet's heat transport characteristics have a wider range of technical applicability. The outcomes of annealing and thinning wires made of copper are measured by the efficiency of heat transfer on the developing consistent surface, which grows exponentially with evolving temperature and velocity variation. During such methods, the kinematics of stretching and the concurrent cooling or heating considerably impact the eminence of the final products. One real-world application of an exponentially shrinking or stretching sheet is in materials engineering and manufacturing, particularly in the production of micro and nanoscale devices. By manipulating the dimensions of a sheet exponentially, it is possible to create structures with precise and controlled variations in size. Overall, the use of exponentially shrinking or stretching sheets provides a versatile approach for fabricating structures with precise and controlled variations in size. These techniques find applications in various fields, including microelectronics, optics, biotechnology, and advanced materials research. Khan and Sanjayanand^5^ studied a second-grade fluid's steady flow and heat conductivity over increasing exponentially surfaces employing the Runge–Kutta fourth-order (RK4) technique. Rehman et al.^6^ explored the stable flow of a second-grade fluid across an exponentially stretched sheet employing the Keller-box technique and the HAM. Nadeem et al.^7^ examined the flow and transmission of the heat of second-grade (viscoelastic) fluid through an exponentially expanding sheet in an environment of thermal radiation. Ramzan and Bilal^8^ studied time-varying MHD, thermal radiation, and mixed convection and conduction flow on the stretched surface in a second-grade nanofluid. Through perturbations assessment, Pakdemirli et al.^9^ evaluated the features of second-grade fluid. Over the past few decades, there has been a lot of investigation on the second-grade nanofluid over an exponentially extending surface^10–15^.

Scientists and technicians appreciate a chaotic flow in several industries because it gives them more significant influence over their decisions^16^. Moreover, even in the most effective flow scenarios, an undesirable insecure impact can appear encircling a structure. The behaviour of unstable BL flow is different from that of regular flow because of the inclusion of additional time-dependent aspects of the mathematical models that influenced the boundary layer (BL) segregation and the movement of the fluid pattern. Still, modern engineering techniques that enable improved reliability, effectiveness, and economics for multiple dynamic components are feasible via an adequate assessment of the unstable flow of fluid implications for production processes^17^. Zaib et al.^18^ described the computational investigation of a time-dependent flow with heat flux past an exponentially contracting surface.

Thermal radiation is a phenomenon that causes heat transmission through electromagnetic waves. It develops since there is significant variation in temperature across the two mediums. Radiative influences are significant in the engineering profession and sciences. In the polymer manufacturing sectors, where heat-controlling variables influence the ultimate product quality to some extent, thermal radiation impacts are essential in controlling heat transfer. Moreover, weapons of mass destruction, planes, combustion engines, solar radiations, spacecraft, metallic liquid fluids, MHD accelerators, and nuclear plants have significant radiative implications. Pantokratoras and Fang^19^ were the inaugural researchers to look at the impacts of nonlinear radiation from nonlinear sources on the Sakiadis flow. Dogonchi and Ganji^20^ tested the influence of radiant heat on the MHD flow over divergent/convergent channels. Khan et al.^21^ studied the electromagnetic radiation propagation of hybrid nanofluid technology throughout an opaque surface. Non-linear thermal radiation has captured the attention of multiple investigators^22–25^.

Recognizing the need for improved thermal conductivity in traditional fluids, a new type of nanofluid called “hybrid nanofluid” is presented to provide highly industrialized heat conductivity. Two or more semiconductor materials are mixed with a base fluid to make a hybrid nanofluid. Different nanomaterials include carbon nanotubes^26^, metals, metal oxides, and carbides. Numerous investigators are now interested in hybrid nanofluid due to its significance for the betterment of thermodynamic characteristics in real-world applications^27^, as a result of Choi and Eastman's^28^ outstanding findings that gave the unique notion of nano liquid. Hybrid nanofluids are also used in various applications, including electrical gadget cooling^29^, cooling of domestic refrigerators^30^, automobile braking fluid, transformers, heat exchangers, and solar water heating^31^. Suresh et al.^32^ explored the effects of a hybrid $\left( {{\text{Al}}_{{2}} {\text{O + Cu/Water}}} \right)$ nanofluid in a circular tube that was uniformly heated. Momin^33^ investigated the thermal act of a hybrid nanofluid in a spherical tube and demonstrated that the hybrid $\left( {{\text{Al}}_{{2}} {\text{O + Cu/Water}}} \right)$ nanofluid improves thermal conductivity compared to a conventional working liquid. Waini et al.^34^ scrutinized the influence of buoyancy on hybrid $\left( {{\text{Al}}_{{2}} {\text{O + Cu/Water}}} \right)$ nanofluid toward the stagnation point of an exponentially stretching/shrinking vertical sheet. They determined that $\left( {{\text{Al}}_{{2}} {\text{O + Cu/Water}}} \right)$ had a higher heat transfer rate than Cu/water. Khan^35^ numerically examined the convection of copper (Cu + Water) and $\left( {{\text{Al}}_{{2}} {\text{O/Water}}} \right)$ nano-liquid across a spinning disc in a porous media. Cu-water has a faster heat transfer rate than Al2O3-water, and the presence of porous media raised the thickness of the thermal BL. Takabi and Salehi^36^ analyzed the heat transfers of ${\text{Al}}_{{2}} {\text{O/Water}}$ and $\left( {{\text{Al}}_{{2}} {\text{O + Cu/Water}}} \right)$ nanofluids with a heat source. The literature is well-stocked with further information on this topic^37–41^.

To reduce wasted energy, the research community has made extraordinary efforts. They were going through energy-converting devices again, coming up with new strategies and approaches to use better what they had. The amount of energy that does not accomplish productive work is called entropy generation. The ratio of recovered heat to temperature is defined as entropy generation. They measured the performance of thermal types of machinery such as heat pumps, electronics, power plants, air conditioners, and energy gadgets employing entropy production. Bejan^42^ utilized entropy optimization to show how thermal performance in fluids behaves. Butta et al.^43^ conducted a theoretical study of the entropy implications of a Casson nanofluid flow across an unsteady stretching surface. They observed that unpredictability aided the creation of entropy effects. The entropy generation in an MHD viscoelastic (Second-grade) fluid past a linearly stretched sheet was investigated by Aiboud and Saouli^44^. Afridi and Qasim^45^ investigated the entropy effects of a 3D Viscous liquid across an exponential sheet. Butta et al.^46^ investigated the entropy generated within a mixed convective magnetohydrodynamic flow of a viscoelastic fluid over a stretching sheet. They observed that unpredictability aided the creation of entropy effects. Dalir et al.^47^ demonstrated entropy generation by a viscoelastic nano-liquid against a stretchable layer. Sithole et al.^48^ use a stretchable surface with viscous dissipation to examine irreversibility in the electromagnetic flow of second-grade nanoparticles. Some relevant entropy optimization efforts are described in^49–51^.

Fuzzy set theory (FST)^52^ has proved to be a valuable technique for modelling uncertainties in recent decades, providing models with a more accurate view of reality and allowing them to express themselves with a wider perspective^53,54^. After modelling real-world problems, they convert into PDEs or ODEs. Uncertainty issues may arise during the development of a dynamic model. Researchers must deal with inaccurate data, parameters, dynamical variability, and complex relationships. As a result, many scientists use fuzzy models to depict dynamical systems to prevent artificial data accuracy and produce more realistic results. The FDE is critical in overcoming these challenges. Chang and Zadeh^55^ proposed the basic idea of fuzzy derivatives. Dubois and Prade^56^ proposed the idea of fuzzy numbers (FNs) for solving an FDE. Kaleva^57^ introduced the concept of FDEs in a fuzzy environment. Recently, FDEs played a significant part in the research field of fluid dynamics, such as when Nadeem et al.^58^ systematically studied the influence of MHD and gravitation on the third-grade fluid across an inclined channel in an environment that is fuzzy. The triangular membership function (MF) was applied to analyze the degree of indeterminacy. Siddiqui et al.^59^ explored the heat transmission of SWCNTs MWCNTS on third-grade nanofluid down an inclined channel in a fuzzy environment. They used the volume fraction of nanoparticles as TFN for comparison and uncertainty. The effect of fuzzy volume fraction on Jeffery-Hamel nanofluid across inclined plates was studied by Biswal et al.^60^. Some current studies relating to the FDEs are included in^61–64^.

On the other hand, a comprehensive examination of the previously cited literature exposes substantial discrepancies and drawbacks. No preceding works have investigated entropy generation due to unsteady MHD flow in second-grade hybrid nanofluid over the exponentially stretching/shrinking sheet with non-linear thermal radiation and fluid dissipation in their investigation structure, according to the authors' understanding. Furthermore, the nanoparticle volume percentages for each nanofluid and a hybrid nanofluid are determined as fuzzy triangular numbers utilizing the dual parametric concept for comparisons and instability. The homotopy analysis technique was used to tackle the problem under consideration. The impact of essential parameters on heat and flow properties and entropy generation are graphed and briefly discussed. This creative approach can help in the development of industrial production, particularly in the areas of engineering and fabrication. We will be able to answer the following research questions once we have solved this problem:

- What causes a decline in the nanofluid and hybrid nanofluid velocity profile when using the magnetic effect accelerates Lorentz forces?
- What are the differences in transport behaviours between non-Newtonian (second-grade fluid) and hybrid nanofluid?
- How does the entropy generation behave concerning different flow parameters?
- How do the fuzzy phenomena affect the temperature of nanofluids and hybrid nanofluids?
- Why does the rise in the magnetic field, non-linear thermal radiation and the nanoparticle volume percentage cause the nanofluid temperature to elevate?
- How do the Nusselt number and drag force respond to different flow parameters?

## Mathematical model

**Figure 1:**
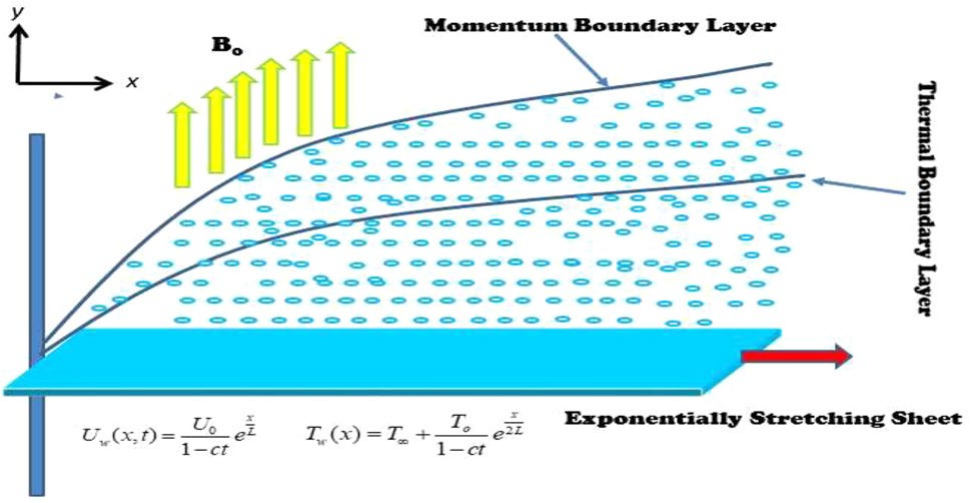
Flow problem.

This study takes into account the time-dependent, 2D incompressible flow of MHD viscoelastic (Second-grade) hybrid ${\text{Al}}_{{2}} {\text{O}}_{{3}} + {\text{Cu/SA}}$ nanofluid over the exponentially shrinking/stretching surface, as shown in Fig. 1. $u_{w} \left( {x,\,t} \right) = \lambda e^{{{x \mathord{\left/ {\vphantom {x L}} \right. \kern-0pt} L}}} \left( {a/\left( {1 - ct} \right)} \right)$ signifies the stretching/shrinking velocity, where $\lambda$ represents a constant that relates to stretching $\lambda > 0$ and shrinking $\lambda < 0$ cases of the velocity rate, *L* indicates the characteristic length, and *c* denotes the unsteadiness. $v_{w} \left( {x,\,t} \right) = \left( {\nu_{o} e^{{{x \mathord{\left/ {\vphantom {x {2L}}} \right. \kern-0pt} {2L}}}} /\sqrt {1 - ct} } \right)$ represents the mass flux velocity, where $v_{o}$ is constant. The variation in temperature near the surface is determined by $T_{w} = T_{\infty } + e^{{{x \mathord{\left/ {\vphantom {x {2L}}} \right. \kern-0pt} {2L}}}} \left( {T_{o} /\left( {1 - ct} \right)} \right)$, the ambient and reference temperatures, denoted as $T_{\infty }$ and $T_{w} ,$ respectively. Also, the fluid at the surface is thought to contain a wall temperature higher than the ambient level. The magnetic field is supposed to be uniform $B\left( t \right) = \left( {B_{o} /\left( {1 - ct} \right)^{0.5} e^{{{x \mathord{\left/ {\vphantom {x {2L}}} \right. \kern-0pt} {2L}}}} } \right),$ with $B_{o}$, denoting a magnetic field, and there is no pressure gradient on the surface. The vicious, source/sink, and nonlinear thermal radiation effects are also considered.

1 $${\hat{\mathbf{S}}} = - p{\mathbf{I}} + \mu {\mathbf{A}}_{{\mathbf{1}}} + \alpha_{1} {\mathbf{A}}_{{\mathbf{2}}} + \alpha_{2} {\mathbf{A}}_{{\mathbf{1}}}^{{\mathbf{2}}} ,$$

2 $$\left. \begin{gathered} {\mathbf{A}}_{1} = \left( {{\text{grad}}{\mathbf{V}}} \right)^{T} {\mathbf{A}}_{0} + {\mathbf{A}}_{0} \left( {{\text{grad}}{\mathbf{V}}} \right), \hfill \\ {\mathbf{A}}_{2} = \frac{{d{\mathbf{A}}_{1} }}{dt} + \left( {{\text{grad}}{\mathbf{V}}} \right)^{T} {\mathbf{A}}_{1} + {\mathbf{A}}_{1} \left( {{\text{grad}}{\mathbf{V}}} \right), \hfill \\ \end{gathered} \right\}$$

where $\alpha_{1} ,\,\,{\text{and}}\,\,\alpha_{2}$ are material constants, where $p$ stands for pressure, ${\hat{\mathbf{S}}}$ is Cauchy tensor, $\mu$ is the dynamic viscosity, **I** is an identity matrix, ${d \mathord{\left/ {\vphantom {d {dt}}} \right. \kern-0pt} {dt}}$ is material time derivative $T$ indicates the matrix transpose, ${\mathbf{A}}_{1} ,\,\,{\text{and}}\,\,{\mathbf{A}}_{2}$ are the first two Revilin–Erickson tensors^1–4^.

When using the BL approximation, the following assumptions are used in calculating the governing equations for continuity, momentum, and heat:^12,48^

3 $$\frac{\partial u}{{\partial x}} + \frac{\partial v}{{\partial y}} = 0,$$

4 $$\frac{\partial u}{{\partial t}} + u\frac{\partial u}{{\partial x}} + v\frac{\partial u}{{\partial y}} = \frac{{\mu_{hnf} }}{{\rho_{hnf} }}\frac{{\partial^{2} u}}{{\partial y^{2} }} + \frac{{\alpha_{1} }}{{\rho_{hnf} }}\left( {\frac{{\partial^{3} u}}{{\partial t\partial y^{3} }} + u\frac{{\partial^{3} u}}{{\partial x\partial y^{3} }} + \frac{\partial u}{{\partial x}}\frac{{\partial^{2} u}}{{\partial y^{2} }} + \frac{\partial u}{{\partial y}}\frac{{\partial^{2} v}}{{\partial y^{2} }} + v\frac{{\partial^{3} u}}{{\partial y^{3} }}} \right) - \frac{{\sigma_{hnf} }}{{\rho_{hnf} }}B_{0}^{2} u,$$

5 $$\begin{aligned} \frac{\partial T}{{\partial t}} + u\frac{\partial T}{{\partial x}} + v\frac{\partial T}{{\partial y}} & = \alpha_{hnf} \frac{{\partial^{2} T}}{{\partial y^{2} }} + \frac{{Q_{0} \left( {T - T_{\infty } } \right)}}{{\left( {\rho c_{P} } \right)_{hnf} }} + \frac{{16\delta^{**} }}{{3\left( {\rho c_{p} } \right)_{trhnf} k^{**} }}\left( {T^{3} \frac{{\partial^{2} T}}{{\partial y^{2} }} + 3T^{2} \left( {\frac{\partial T}{{\partial y}}} \right)^{2} } \right) \\ & \quad + \frac{{\mu_{hnf} }}{{\left( {\rho c_{P} } \right)_{hnf} }}\left( {\frac{\partial u}{{\partial y}}} \right)^{2} + \frac{{\alpha_{1} }}{{\left( {\rho c_{P} } \right)_{hnf} }}\left( {\frac{\partial u}{{\partial y}}\frac{{\partial^{2} u}}{\partial y\partial t} + u\frac{\partial u}{{\partial y}}\frac{{\partial^{2} u}}{\partial x\partial y} + v\frac{\partial u}{{\partial y}}\frac{{\partial^{2} u}}{{\partial y^{2} }}} \right), \\ \end{aligned}$$

the boundary conditions are:

6 $$\left. \begin{gathered} t < 0:\,\,\,\,T = T_{\infty } ,\,\,\,\,v = 0,\,\,\,\,u = 0,\,\,\,\,\,\,\,\,\,\forall \,x,\,y, \hfill \\ t \ge 0:\,\,\,\,v = v_{w} ,\,\,\,\,u = U_{w} \left( {x,\,t} \right) = u_{w} \left( {x,\,t} \right),\,\,\,\,T = T_{w} \,\,\,\,\,\,{\text{at}}\,\,y \to 0, \hfill \\ \,\,\,\,\,\,\,\,\,\,\,\,\,\,\,\,\,\,\,u = 0,\,\,\,\,T = T_{\infty } \,\,\,\,\,\,\,\,\,\,\,\,\,\,\,\,\,\,\,\,\,\,\,\,\,\,\,\,\,\,\,\,\,\,\,\,\,\,\,\,\,\,\,\,\,\,\,\,\,\,\,\,\,\,\,\,\,\,\,{\text{as}}\,\,\,\,y \to \infty , \hfill \\ \end{gathered} \right\}$$

where $u$ and $v$ are the velocity components along the $x{\text{-a}\overline{\text{x}}\text{is}}$ and $y{\text{-a}\overline{\text{x}}\text{is,}}$ respectively, and *T* shows the fluid temperature. The sheet surface's suction and injection velocities are assumed to be distributed. The dimensionless wall mass transfer coefficient is denoted by $v_{w}$ here. It displays the mass injection process when we select a velocity of $v_{w} > 0$ across the boundary wall's surface, and the mass suction process when we select a velocity of mass $v_{w} < 0.$ The dynamic viscosity of ${\text{Al}}_{{2}} {\text{O}}_{{3}} + {\text{Cu/SA}}$ is $\mu_{hnf} ,$ $\left( {\rho Cp} \right)_{hnf}$ is the ${\text{Al}}_{{2}} {\text{O}}_{{3}}+ {\text{ Cu/SA}}$ heat capacity, $k_{hnf}$ is the ${\text{Al}}_{{2}} {\text{O}}_{{3}} + {\text{Cu/SA}}$ thermal/heat conductivity, $\rho_{hnf}$ the density of ${\text{Al}}_{{2}} {\text{O}}_{{3}} + {\text{Cu/SA}}$, and $\delta_{hnf}$ the electrical conductivity ${\text{Al}}_{{2}} {\text{O}}_{{3}} + {\text{Cu/SA}}$. The aluminium oxide $\left( {{\text{Al}}_{{2}} {\text{O}}_{{3}} } \right)$ thermophysical properties, along with copper (Cu) and Sodium alginate (SA) nanoparticles, are revealed in Table 1. Equation 8 contains the thermophysical properties of ${\text{Al}}_{{2}} {\text{O}}_{{3}} + {\text{Cu/SA}}$. Here $\phi_{1}$ and $\phi_{2}$ are volume fractions of ${\text{Al}}_{{2}} {\text{O}}_{{3}}$ and ${\text{Cu}}$ respectively. Further, if $\alpha_{1} = 0$, then, Eq. 4 is converted into a Newtonian fluid.

**Table 1:** The ${\text{Al}}_{{2}} {\text{O}}_{{3}}$ thermo-physical properties along with ${\text{Cu}}$ and SA at 20 °C (293 K)^63^.

Physical properties	*ρ* (kg/m^3^)	*ρc*_*p*_ (J/kgK)	*k* (W/mK)	$\beta_{T} \times 10^{ - 5} \;\left( {1/{\text{K}}} \right)$	*σ* (Ω/m)^−1^
SA	989	4175	0.6376	99	$2.6 \times 10^{ - 4}$
${\text{Al}}_{{2}} {\text{O}}_{{3}}$	3970	765	40	0.85	$3.69 \times 10^{7}$
${\text{Cu}}$	8933	385	401	1.67	$5.96 \times 10^{7}$

According to the Roseland approximation for radiation in Eq. (7), $q_{r}$ is the radiative heat flow is defined as follows:

7 $$\left. \begin{gathered} q_{r} = - \frac{{4\delta^{**} }}{{3k^{**} }}\frac{{\partial T^{4} }}{\partial y}\,\, = - \frac{{16\delta^{**} T^{3} }}{{3k^{**} }}\frac{\partial T}{{\partial y}} \hfill \\ \Rightarrow \frac{{\partial q_{r} }}{\partial y} = - \frac{{16\delta^{**} }}{{3k^{**} }}\left( {T^{3} \frac{{\partial^{2} T}}{{\partial y^{2} }} + 3T^{2} \left( {\frac{\partial T}{{\partial y}}} \right)^{2} } \right), \hfill \\ \end{gathered} \right\}$$

in which the $$k^{**}$$ and $$\delta^{**}$$ are the delegate of the mean absorption coefficient and Stefan–Boltzman constant, respectively.

The thermophysical properties of the hybrid nanofluids are^63^:

8 $$\left. \begin{gathered} \rho_{r} = \frac{{\rho_{hnf} }}{{\rho_{f} }} = \left[ {\left( {1 - \phi_{2} } \right)\left\{ {\left( {1 - \phi_{1} } \right) + \frac{{\rho_{{s_{1} }} \phi_{1} }}{{\rho_{f} }}} \right\} + \frac{{\rho_{{s_{2} }} \phi_{2} }}{{\rho_{f} }}} \right],\mu_{r} = \frac{{\mu_{hnf} }}{{\mu_{f} }} = \left( {1 - \phi_{1} } \right)^{ - 2.5} \left( {1 - \phi_{2} } \right)^{ - 2.5} , \hfill \\ \left( {\rho C_{\rho } } \right)_{r} = \frac{{\left( {\rho C_{\rho } } \right)_{hnf} }}{{\left( {\rho C_{\rho } } \right)_{f} }} = \frac{{\phi_{2} \left( {\rho C_{\rho } } \right)_{{s_{2} }} }}{{\left( {\rho C_{\rho } } \right)_{f} }} + \left( {1 - \phi_{2} } \right)\left[ {\left( {1 - \phi_{1} } \right) + \frac{{\left( {\rho C_{\rho } } \right)_{{s_{1} }} \phi_{1} }}{{\left( {\rho C_{\rho } } \right)_{f} }}} \right],\alpha_{hnf} = \frac{{k_{hnf} }}{{\left( {\rho c_{P} } \right)_{hnf} }}, \hfill \\ k_{r} = \frac{{k_{hnf} }}{{k_{nf} }} = \frac{{2k_{nf} - 2\phi_{1} \left( {k_{{s_{1} }} - k_{nf} } \right) + k_{{s_{1} }} }}{{2k_{nf} + \phi_{1} \left( {k_{{s_{1} }} - k_{nf} } \right) + k_{{s_{1} }} }},\frac{{k_{nf} }}{{k_{f} }} = \frac{{2k_{f} - 2\phi_{2} \left( {k_{{s_{2} }} - k_{f} } \right) + k_{{s_{2} }} }}{{2k_{f} + \phi_{2} \left( {k_{{s_{2} }} - k_{f} } \right) + k_{{s_{2} }} }}, \hfill \\ \sigma_{r} = \frac{{\sigma_{hnf} }}{{\sigma_{bf} }} = \left[ {\frac{{\sigma_{{s_{2} }} \left( {1 + 2\phi_{2} } \right) + 2\sigma_{bf} \left( {1 - \phi_{2} } \right)}}{{\sigma_{{s_{2} }} \left( {1 - \phi_{2} } \right) + \sigma_{bf} \left( {2 + \phi_{2} } \right)}}} \right],\sigma_{bf} = \left[ {\frac{{\sigma_{{s_{1} }} \left( {1 + 2\phi_{1} } \right) + 2\sigma_{f} \left( {1 - \phi_{1} } \right)}}{{\sigma_{{s_{1} }} \left( {1 - \phi_{1} } \right) + \sigma_{f} \left( {2 + \phi_{1} } \right)}}} \right]\sigma_{f} . \hfill \\ \end{gathered} \right\}$$

To clarify Eqs. (3)–(6), the subsequent similarity transformations are presented^10^ in a considerably simpler method, where the stream's function $\omega$ is adjustable as $v = - {{\partial \omega } \mathord{\left/ {\vphantom {{\partial \omega } {\partial x,\,\,\,u = {{\partial \omega } \mathord{\left/ {\vphantom {{\partial \omega } {\partial y,\,\,}}} \right. \kern-0pt} {\partial y,\,\,}}\,\,}}} \right. \kern-0pt} {\partial x,\,\,\,u = {{\partial \omega } \mathord{\left/ {\vphantom {{\partial \omega } {\partial y,\,\,}}} \right. \kern-0pt} {\partial y,\,\,}}\,\,}}$ and its similarity variable is $\eta :$

9 $$\left. \begin{gathered} \omega = \sqrt {\frac{{2la\nu_{f} }}{1 - ct}} e^{{{\raise0.7ex\hbox{$x$} \!\mathord{\left/ {\vphantom {x {2L}}}\right.\kern-0pt} \!\lower0.7ex\hbox{${2L}$}}}} f\left( \eta \right),\,\,\,\,\eta = \sqrt {\frac{a}{{2l\nu_{f} \left( {1 - ct} \right)}}} e^{{{\raise0.7ex\hbox{$x$} \!\mathord{\left/ {\vphantom {x {2L}}}\right.\kern-0pt} \!\lower0.7ex\hbox{${2L}$}}}} y,\,\,\,\theta \left( \eta \right) = \frac{{T - T_{\infty } }}{{T_{w} - T_{\infty } }}, \hfill \\ u = \frac{{ae^{{{\raise0.7ex\hbox{$x$} \!\mathord{\left/ {\vphantom {x L}}\right.\kern-0pt} \!\lower0.7ex\hbox{$L$}}}} }}{1 - ct}f^{\prime}\left( \eta \right),\,\,\,v = - \sqrt {\frac{{a\nu_{f} }}{{2l\left( {1 - ct} \right)}}} e^{{{\raise0.7ex\hbox{$x$} \!\mathord{\left/ {\vphantom {x {2L}}}\right.\kern-0pt} \!\lower0.7ex\hbox{${2L}$}}}} \left( {f\left( \eta \right) + \eta f^{\prime}\left( \eta \right)} \right). \hfill \\ \end{gathered} \right\}$$

Considering Eq. (9), the subsequent nonlinear ODEs Eqs. (3)–(6) are capable of being condensed in the framework of the abovementioned links:

10 $$\begin{aligned} & \left( {\frac{{\mu_{r} }}{{\rho_{r} }}} \right)f^{\prime\prime\prime} + \frac{\alpha }{{\rho_{r} }}\left( {\beta f^{\prime\prime\prime} - \left( {f^{\prime\prime}} \right)^{2} + 2f^{\prime}f^{\prime\prime\prime} - ff^{iv} + \frac{\beta \eta }{2}f^{iv} } \right) - 2\left( {f^{\prime}} \right)^{2} - \beta \left( {f^{\prime} + \frac{\eta }{2}f^{\prime\prime}} \right) + ff^{\prime\prime} \\ & \quad - \frac{{\sigma_{r} }}{{\rho_{r} }}Mf^{\prime} = 0, \\ \end{aligned}$$

11 $$\begin{aligned} & \alpha_{r} \theta^{\prime\prime} + \Pr f\theta^{\prime} - \Pr \theta f^{\prime} - \Pr \beta \left( {\frac{1}{2}\eta \theta^{\prime} + \theta } \right) + \frac{{\alpha {\text{PrEc}} }}{{\left( {\rho c_{P} } \right)_{r} }}f^{\prime\prime}\left( {2\eta f^{\prime\prime} - ff^{\prime\prime\prime} + 2f^{\prime}f^{\prime\prime} + \eta \beta f^{\prime\prime\prime}} \right) + \frac{{{\text{PrH}} \theta }}{{\left( {\rho c_{P} } \right)_{r} }} \\ & \quad + \frac{{\mu_{r} {\text{PrEc}} }}{{\left( {\rho c_{P} } \right)_{r} }}\left( {f^{\prime\prime}} \right)^{2} = 0, \\ \end{aligned}$$

with the constraints

12 $$\,\left. \begin{array}{llllll} f\left( \eta \right) = s,\,\,\,\,f^{\prime}\left( \eta \right) = \lambda ,\,\,\,\,\theta \left( \eta \right) = 1\,\,\,\,\,\,{\text{at}}\,\,\,\,\,\,\,\eta \, = \,0, \hfill \\ f^{\prime}\left( \eta \right) = 0,\,\, \,\theta \left( \eta \right) = 0\,\,\,\,\,\, {\text{at}}\,\,\,\, \eta \, \to \infty ,\,\,\, \hfill \\ \end{array} \right\}$$

where the unsteadiness parameter is $$\beta = {{2Lc} \mathord{\left/ {\vphantom {{2Lc} {ae^{{{\raise0.7ex\hbox{$x$} \!\mathord{\left/ {\vphantom {x L}}\right.\kern-0pt} \!\lower0.7ex\hbox{$L$}}}} }}} \right. \kern-0pt} {ae^{{{\raise0.7ex\hbox{$x$} \!\mathord{\left/ {\vphantom {x L}}\right.\kern-0pt} \!\lower0.7ex\hbox{$L$}}}} }},$$ the magnetic parameter is $$M = {{2\delta_{f} LB_{o}^{2} } \mathord{\left/ {\vphantom {{2\delta_{f} LB_{o}^{2} } {a\rho_{f} }}} \right. \kern-0pt} {a\rho_{f} }},$$ the second-grade fluid parameter is $$\alpha = {a \mathord{\left/ {\vphantom {a {2L\mu_{f} \left( {1 - ct} \right)}}} \right. \kern-0pt} {2L\mu_{f} \left( {1 - ct} \right)}},$$ the Prandtl number is $$\Pr = {{\nu_{f} } \mathord{\left/ {\vphantom {{\nu_{f} } {\alpha_{f} }}} \right. \kern-0pt} {\alpha_{f} }},$$ the Eckert number is $$Ec = {{a^{2} } \mathord{\left/ {\vphantom {{a^{2} } {\left( {1 - ct} \right)^{2} \left( {T_{w} - T_{\infty } } \right)\left( {C_{\rho } } \right)_{f} }}} \right. \kern-0pt} {\left( {1 - ct} \right)^{2} \left( {T_{w} - T_{\infty } } \right)\left( {C_{\rho } } \right)_{f} }},$$ the heat generation/absorption parameter is $$H = {{2L\left( {1 - ct} \right)\left( {T_{w} - T_{\infty } } \right)Q_{o} } \mathord{\left/ {\vphantom {{2L\left( {1 - ct} \right)\left( {T_{w} - T_{\infty } } \right)Q_{o} } a}} \right. \kern-0pt} a},$$ the suction parameter is $$s = - v_{o} \sqrt {{{2L} \mathord{\left/ {\vphantom {{2L} {a\nu_{f} }}} \right. \kern-0pt} {a\nu_{f} }}}$$ and the stretching/shrinking parameter is $$\lambda .$$ The coefficient of skin friction $$\left( {C_{fx} } \right)$$ and the local Nusselt number $$\left( {{\text{Nu}}_{{\text{x}}} } \right)$$ are thus demarcated as:

13 $$C_{fx} = \frac{1}{{\rho_{f} u_{e}^{2} }}\left[ {\mu_{hnf} \frac{\partial u}{{\partial y}} + \alpha_{1} \left\{ {u\frac{{\partial^{2} u}}{\partial x\partial y} + v\frac{{\partial^{2} u}}{{\partial y^{2} }} + \frac{{\partial^{2} u}}{\partial t\partial y} + 2\frac{\partial u}{{\partial y}}\frac{\partial u}{{\partial x}}} \right\}} \right]_{y = 0} .$$

14 $$Nu_{x} = - \frac{x}{{k_{f} \left( {T_{w} - T_{\infty } } \right)}}\left[ {k_{hnf} \frac{\partial u}{{\partial y}} + \frac{{16\sigma^{*} T_{\infty }^{3} }}{{3k^{*} }}\frac{\partial u}{{\partial y}}} \right]_{y = 0} .$$

Then use (9) into (13) and (14), which produces an apparent connection:

15 $$\sqrt {{\text{Re}}_{x} } C_{fx} = \left( {\mu_{r} f^{\prime\prime}\left( 0 \right) + \alpha \left( {3f^{\prime}\left( 0 \right)f^{\prime\prime}\left( 0 \right) - f\left( 0 \right)f^{\prime\prime\prime}\left( 0 \right) + \frac{3\beta }{2}f^{\prime\prime}\left( 0 \right) + \frac{\beta }{2}\eta f^{\prime\prime\prime}\left( 0 \right)} \right)} \right),$$

16 $$\left( {{\text{Re}}_{x} } \right)^{ - 0.5} Nu_{x} = - \left( {k_{r} + Nr\left( {1 + \theta \left( 0 \right)\left( {\theta_{w} - 1} \right)} \right)^{3} } \right)\theta^{\prime}\left( 0 \right),$$

where $Re_{x} = {{u_{e} x} \mathord{\left/ {\vphantom {{u_{e} x} {\nu_{f} }}} \right. \kern-0pt} {\nu_{f} }}$ is the *x*-axis local Reynolds number.

### Entropy analysis

Entropy generation is a concept used in thermodynamics and fluid dynamics to quantify the irreversibility and losses that occur within a system. It measures the inefficiency or degradation of energy within a process. Expert's concerns about energy management in any experiment or study were sufficient to prevent valuable energy from vanishing. Entropy formation analysis might be more efficient for carrying out this crucial activity. This allows doctors to discover issues such as MHD impacting its irreversibility. While implementing the boundary layer approximation, the equation for the local volumetric rate of entropy development in the presence of a magnetic field for the second-grade hybrid nanofluid is^48^:

17 $$\begin{aligned} S_{G} & = \frac{{k_{hnf} }}{{T_{\infty }^{2} }}\left( {\frac{\partial T}{{\partial y}}} \right)^{2} + \frac{1}{{T_{\infty } }}\left[ {\frac{{\alpha_{1} }}{{T_{\infty } }}\left( {\frac{\partial u}{{\partial y}}\frac{{\partial^{2} u}}{\partial y\partial t} + u\frac{\partial u}{{\partial y}}\frac{{\partial^{2} u}}{\partial x\partial y} + v\frac{\partial u}{{\partial y}}\frac{{\partial^{2} u}}{{\partial y^{2} }}} \right) + \mu_{hnf} \left( {\frac{\partial u}{{\partial y}}} \right)^{2} } \right] \\ & \quad + \frac{{k_{hnf} }}{{T_{\infty }^{2} }}\frac{{16\delta^{*} T_{\infty }^{3} }}{{3k^{*} }}\left( {\frac{\partial T}{{\partial y}}} \right)^{2} + \frac{{\delta_{hnf} B_{o}^{2} }}{{T_{\infty } }}u^{2} , \\ \end{aligned}$$

here viscous dissipation, heat transmission, and magnetic field are all crucial elements in the formation of entropy effects, as seen in the above formula.

Equation (9) can be used to construct the dimensionless form of Eq. (16)'s formula for local entropy generation:

18 $$\begin{aligned} N_{S} & = A{\text{Re}} \left( {\theta^{\prime}} \right)^{2} \left( {k_{r} + Nr\left( {1 + \left( {\theta_{w} - 1} \right)\theta } \right)^{3} } \right) + {\text{ReBr}} \alpha f^{\prime\prime}\left[ {\mu_{r} f^{\prime\prime} + 3\beta f^{\prime\prime} + 3f^{\prime}f^{\prime\prime} + \beta f^{\prime\prime\prime} - ff^{\prime\prime\prime}} \right] \\ & \quad + \delta_{r} MBr{\text{Re}} \left( {f^{\prime}} \right)^{2} , \\ \end{aligned}$$

here, the dimensionless temperature gradient is $A = \frac{{T_{w} - T_{\infty } }}{{T_{\infty } }},$ the local entropy generation number is $N_{S} = \frac{{S_{G} T_{\infty } L}}{{k_{f} \left( {T_{w} - T_{\infty } } \right)}},$ and the Brinkman number is $Br = \Pr Ec.$

### Homotopy analysis method (HAM)

The HAM is a sophisticated analytical technique for solving nonlinear equations with many variables. The HAM technique is used to determine the consequent Eqs. (10) and (11) with the help of the boundary conditions (12). To start the process of employing this technique, linear operators and preliminary predictions are essential. Consequently, we utilized the previously mentioned technique to resolve the energy and motion transformation models employing $\left( {\Lambda_{f} ,\,\,\Lambda_{\theta } } \right)$ as linear operators and $\left( {f_{0} \left( \eta \right),\,\,\theta_{0} \left( \eta \right)} \right)$ the initial estimations. See^9–12^ for more information on this technique.

19 $$\left. \begin{gathered} f_{0} \left( \eta \right) = s - \lambda \left( {1 - e^{ - \eta } } \right),\,\,\,\,\,\,\,\,\,\,\,\,\,\,\,\theta_{0} \left( \eta \right) = e^{ - \eta } , \hfill \\ \Lambda_{f} \left[ {f\left( \eta \right)} \right] = f^{\prime\prime\prime} - f^{\prime},\,\,\,\,\,\,\,\,\,\,\,\,\,\,\,\,\Lambda_{\theta } \left[ {\theta \left( \eta \right)} \right] = \theta^{\prime\prime} - \theta . \hfill \\ \end{gathered} \right\}$$

The properties of the operator mentioned above are as follows:

20 $$\left. \begin{gathered} \Lambda_{f} \left( {C_{1} + C_{2} e^{ - \eta } + C_{3} e^{\eta } } \right) = 0, \hfill \\ \Lambda_{\theta } \left( {C_{4} e^{ - \eta } + C_{5} e^{\eta } } \right) = 0, \hfill \\ \end{gathered} \right\}$$

where $C_{js}$ (j = 1, 2, …5) are arbitrary constants.

21 $$\left. \begin{gathered} \left( {1 - q} \right)\Lambda_{f} \left[ {\tilde{F}\left( {\eta ;q} \right) - \tilde{f}_{0} \left( \eta \right)} \right] - qh_{f} N_{f} \left[ {\tilde{F}\left( {\eta ;q} \right)} \right] = 0, \hfill \\ \left( {1 - q} \right)\Lambda_{\theta } \left[ {\tilde{\theta }\left( {\eta ;q} \right) - \tilde{\theta }_{0} \left( \eta \right)} \right] - qh_{\theta } N_{\theta } \left[ {\tilde{F}\left( {\eta ;q} \right),\,\tilde{\theta }\left( {\eta ;q} \right)} \right] = 0, \hfill \\ \end{gathered} \right\}$$

where $h_{f} \,\,{\text{and}}\,\,h_{\theta }$ signify non‐zero auxiliary parameters and $q \in \left[ {0,\,1} \right]$ represents an embedding parameter while $\tilde{F}\,\,\,{\text{and}}\,\,\,\tilde{\theta }$ representing the mapping occupations for $f\,\,{\text{and}}\,\,\theta .$

The boundary conditions become

22 $$\left. \begin{gathered} \tilde{F}\left( {0;q} \right) = s,\,\,\tilde{F}^{\prime}\left( {0;q} \right) = \lambda ,\,\,\,\tilde{\theta }\left( {0;q} \right) = 1,\,\, \hfill \\ \tilde{F}^{\prime}\left( {\infty ;q} \right) = 0,\,\,\,\tilde{\theta }\left( {\infty ;q} \right) = 0. \hfill \\ \end{gathered} \right\}$$

23 $$\begin{aligned} N_{f} \left[ {\tilde{F}\left( {\eta ;q} \right)} \right] & = \left( {\frac{{\mu_{r} }}{{\rho_{r} }}} \right)\frac{{d\tilde{F}^{\prime\prime\prime}\left( {\eta ;q} \right)}}{d\eta } - \beta \left( {\frac{{d\tilde{F}^{\prime}\left( {\eta ;q} \right)}}{d\eta } + \frac{\eta }{2}\frac{{d\tilde{F}^{\prime\prime}\left( {\eta ;q} \right)}}{d\eta }} \right) - 2\left( {\frac{{d\tilde{F}^{\prime}\left( {\eta ;q} \right)}}{d\eta }} \right)^{2} + \frac{{d\tilde{F}\left( {\eta ;q} \right)}}{d\eta }\frac{{d\tilde{F}^{\prime\prime}\left( {\eta ;q} \right)}}{d\eta } \\ & \quad + \frac{\alpha }{{\rho_{r} }}\left( {\beta \frac{{d\tilde{F}^{\prime\prime\prime}\left( {\eta ;q} \right)}}{d\eta } - \left( {\frac{{d\tilde{F}^{\prime\prime}\left( {\eta ;q} \right)}}{d\eta }} \right)^{2} - \tilde{F}\left( {\eta ;q} \right)\frac{{d\tilde{F}^{\prime\prime\prime\prime}\left( {\eta ;q} \right)}}{d\eta } + 2\frac{{d\tilde{F}^{\prime}\left( {\eta ;q} \right)}}{d\eta }\frac{{d\tilde{F}^{\prime\prime\prime}\left( {\eta ;q} \right)}}{d\eta } + \frac{\beta \eta }{2}\frac{{d\tilde{F}^{\prime\prime\prime\prime}\left( {\eta ;q} \right)}}{d\eta }} \right) \\ & \quad - \left( {\frac{{\sigma_{r} }}{{\rho_{r} }}} \right)M\frac{{d\tilde{F}^{\prime}\left( {\eta ;q} \right)}}{d\eta }, \\ \end{aligned}$$

24 $$\begin{aligned} & N_{\theta } \left[ {\tilde{F}\left( {\eta ;q} \right),\tilde{\theta }\left( {\eta ;q} \right)} \right] = \alpha_{r} \left( {1 + Nr\left( {1 + \tilde{\theta }\left( {\eta ;q} \right)\left( {\theta_{w} - 1} \right)} \right)^{3} } \right)\frac{{d\tilde{\theta }^{\prime\prime}\left( {\eta ;q} \right)}}{d\eta } \\ & \quad + 3Nr\left( {\frac{{d\tilde{\theta }^{\prime}\left( {\eta ;q} \right)}}{d\eta }} \right)^{2} \left( {\theta_{w} - 1} \right)\left( {1 + \tilde{\theta }\left( {\eta ;q} \right)\left( {\theta_{w} - 1} \right)} \right)^{2} - \Pr \tilde{\theta }\left( {\eta ;q} \right)\frac{{d\tilde{F}^{\prime}\left( {\eta ;q} \right)}}{d\eta } \\ & \quad + \Pr \tilde{F}\left( {\eta ;q} \right)\frac{{d\tilde{\phi }^{\prime\prime}\left( {\eta ;q} \right)}}{d\eta } + \Pr \tilde{F}\left( {\eta ;q} \right)\frac{{d\tilde{\theta }^{\prime}\left( {\eta ;q} \right)}}{d\eta } + \frac{{\mu_{r} {\text{PrEc}} }}{{\left( {\rho c_{P} } \right)_{r} }}\left( {\frac{{d\tilde{F}^{\prime\prime}\left( {\eta ;q} \right)}}{d\eta }} \right)^{2} \\ & \quad - \Pr \beta \left( {\frac{1}{2}\eta \frac{{d\tilde{\theta }^{\prime}\left( {\eta ;q} \right)}}{d\eta } + \tilde{\theta }\left( {\eta ;q} \right)} \right) + \frac{{\alpha {\text{PrEc}} }}{{\left( {\rho c_{P} } \right)_{r} }}\frac{{d\tilde{F}^{\prime\prime}\left( {\eta ;q} \right)}}{d\eta }\left( \begin{gathered} 2\eta \frac{{d\tilde{F}^{\prime\prime}\left( {\eta ;q} \right)}}{d\eta } + 2\frac{{d\tilde{F}^{\prime}\left( {\eta ;q} \right)}}{d\eta }\frac{{d\tilde{F}^{\prime\prime}\left( {\eta ;q} \right)}}{d\eta } \hfill \\ + \eta \beta \frac{{d\tilde{F}^{\prime\prime\prime}\left( {\eta ;q} \right)}}{d\eta } - \tilde{F}\left( {\eta ;q} \right)\frac{{d\tilde{F}^{\prime\prime\prime}\left( {\eta ;q} \right)}}{d\eta } \hfill \\ \end{gathered} \right), \\ \end{aligned}$$

the Eqs. (10) to (12) convert in the non-linear operators like Eqs. (22–24), then the series solution becomes:

25 $$\left. \begin{gathered} f\left( \eta \right) = f_{0} \left( \eta \right) + \sum\limits_{m = 1}^{\infty } {f_{m} \left( \eta \right)} , \hfill \\ \theta \left( \eta \right) = \theta_{0} \left( \eta \right) + \sum\limits_{m = 1}^{\infty } {\theta_{m} \left( \eta \right)} . \hfill \\ \end{gathered} \right\}$$

### For fuzzification

The governing FDE is converted into a double parametric form. In this case, Eq. (8) can be converted into interval form using the $\chi {\text{-cut}}$ concept. Here $\chi$ and $\omega$ are parameters that range from 0 to 1, controlling the fuzziness of the uncertain parameters. The named problem was solved using the HAM as well. See for further details^53^.

TFNs come in especially handy when you have higher confidence in the center estimate while allowing for some variance and the range of probable values is poorly understood. They offer a straightforward and natural method of modelling and representing uncertainty in decision-making. Using fuzzy concepts, comparing nanofluid and hybrid nanofluid is also explored in this study. The nonlinear ODEs convert into an FDE, and the nanoparticle's volume percentage is a TFN. The slight variation in the volume percentage of nanoparticles impacts the flow rate and heat. These parameters alone determine the nanofluid's flow rate and heat transfer because some researchers estimate that the volume percentage of nanoparticles falls within the [1–4%] range. It is preferable to address a challenging situation in a fuzzy atmosphere by getting both volume fractions as a TFN, since $\phi_{1}$ and $\phi_{2}$ signify the volume fraction of ${\text{Al}}_{{2}} {\text{O}}_{{3}} /{\text{SA}}$ and Cu/SA, separately as shown in Table 2. The volume fractions of nanoparticles used in this study are classified as TFNs, with the TFNs being transformed into $\chi {\text{-cut}}$ methods, and the fuzziness of the TFNs is controlled by $\chi {\text{-cut}}$^61–64^.

**Table 2:** $\phi_{1} \,\,{\text{and}}\,\,\phi_{2}$ transforms into TFN^61–64^.

Fuzzy numbers	Crisp value	TFN	$\chi {\text{-cut}}$ approach
$\phi_{1}$ $\left( {{\text{Al}}_{{2}} {\text{O}}_{{3}} } \right)$	[0.01–0.04]	[0, 0.05, 0.1]	$\left[ {0.05\chi ,\,\,0.1 - 0.05\chi } \right],\,\,\chi \in \left[ {0,\,1} \right]$
$\phi_{2}$ $\left( {{\text{Cu}}} \right)$	[0.01–0.04]	[0, 0.05, 0.1]	$\left[ {0.05\chi ,\,\,0.1 - 0.05\chi } \right],\,\,\chi \in \left[ {0,\,1} \right]$

**Figure 2:**
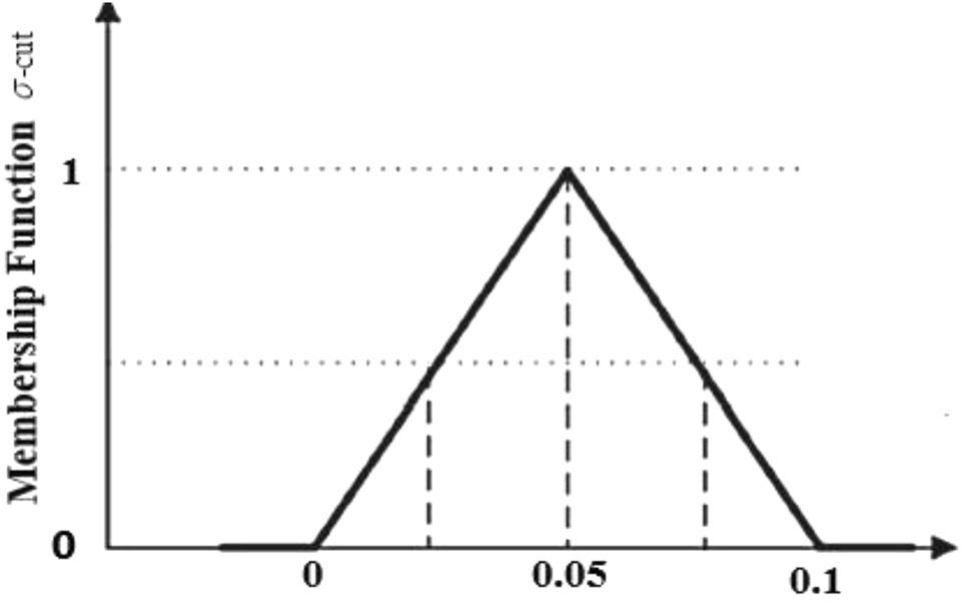
Membership function of TFN^54^.

Let $\phi_{1} = \phi_{2} = \left[ { \, 0,\,\,0.05,\,\,0.1} \right]$ be a TFN that is described by the three values highlighted in Fig. 2: 0 (lower bound), 0.05 (most belief value), and 0.1 (upper bound). The membership function can be represented by the TFN as follows:

26 $${\text{Membership function}} = \left\{ \begin{gathered} \frac{0 - \eta }{{0.05 - 0}}\,\,\,\,\,\,\,\,{\text{for}}\,\,\,\,\,\,\,\eta \in [0,\,\,0.05], \hfill \\ \,\frac{\eta - 0.1}{{0.1 - 0.05}}\,\,\,\,{\text{for}}\,\,\,\,\,\,\eta \in [0.05,\,\,0.1], \hfill \\ \,\,\,\,\,\,0,\,\,\,\,\,\,\,\,\,\,\,\,\,\,\,\,\,\,\,\,\,\,\,\,\,\,\,\,{\text{otherwise}}{.} \hfill \\ \end{gathered} \right.$$

The $$\sigma {\text{-cut}}$$ technique is employed to convert TFNs into interval form and is represented as $$\phi_{1} = \phi_{2} =$$$$\left[ {0 + \chi (0.05 - 0),\,\,0.1 - \chi (0.1 - 0.05)} \right],$$ where $$0 \le \chi {\text{-cut}} \le 1.$$

The FDEs are renewed into lower $\theta_{1} (\eta ,\,\,\chi )$ and upper bounds. $\theta_{2} (\eta ,\,\,\chi ).$ to handle this scenario.

## Results and discussion

An unsteady flow analysis was carried out on the second-grade hybrid nanofluid over an exponential surface. In this situation, a fluid that conducts electricity is studied because it combines viscoelastic and nonlinear radiative heat transmission. Examine the effects of dynamic factors on our system's velocity, temperature, and entropy generation contours. Moreover, an approximation analytical method has been used to control the framework's modified equations. We have acknowledged the most significant characteristics of the simulation we run as $0 \le M \le 1.2$, $0 \le \alpha \le 1.8$, $0 \le \beta \le 1$, $0 \le s \le 1.5$, $0.1 \le \lambda \le 0.7$, $0.01 \le \phi_{1} \le 0.3$, $0.01 \le \phi_{2} \le 0.3$, $0 \le Nr \le 0.6$, $0.8 \le \theta_{w} \le 1.4$, $0 \le H \le 0.45$, $0.1 \le {\text{Re}} \le 1.3$, $0.1 \le A \le 0.4$, $0 \le Br \le 6$, *Pr* = 12 and *Ec* = 0.3.

Table 3 was generated to confirm the numerical results of $\theta^{\prime}\left( 0 \right)$ Haider et al.^51^. The numerical results generated by the HAM in this research have been determined to exist in outstanding agreement with previous research.

**Table 3:** Comparison of current results of $\theta^{\prime}\left( 0 \right)$ Haider et al.^64^ for variation in the *Pr* and *M* when H = 0.0, Nr = 0.0, $\beta = 0.0,$ Ec = 0.0.

M	Pr	Haider et al.^51^ (HAM)	Haider et al.^51^ (NM)	Present (HAM)
0	1	0.95478	0.95478	0.95477
	2	1.47146	1.47146	1.47145
	3	1.86907	1.86907	1.86906
	5	2.50012	2.50012	2.50012
	10	3.66027	3.66027	3.66026
1	1	0.56109	0.56109	0.56108

**Figure 3:**
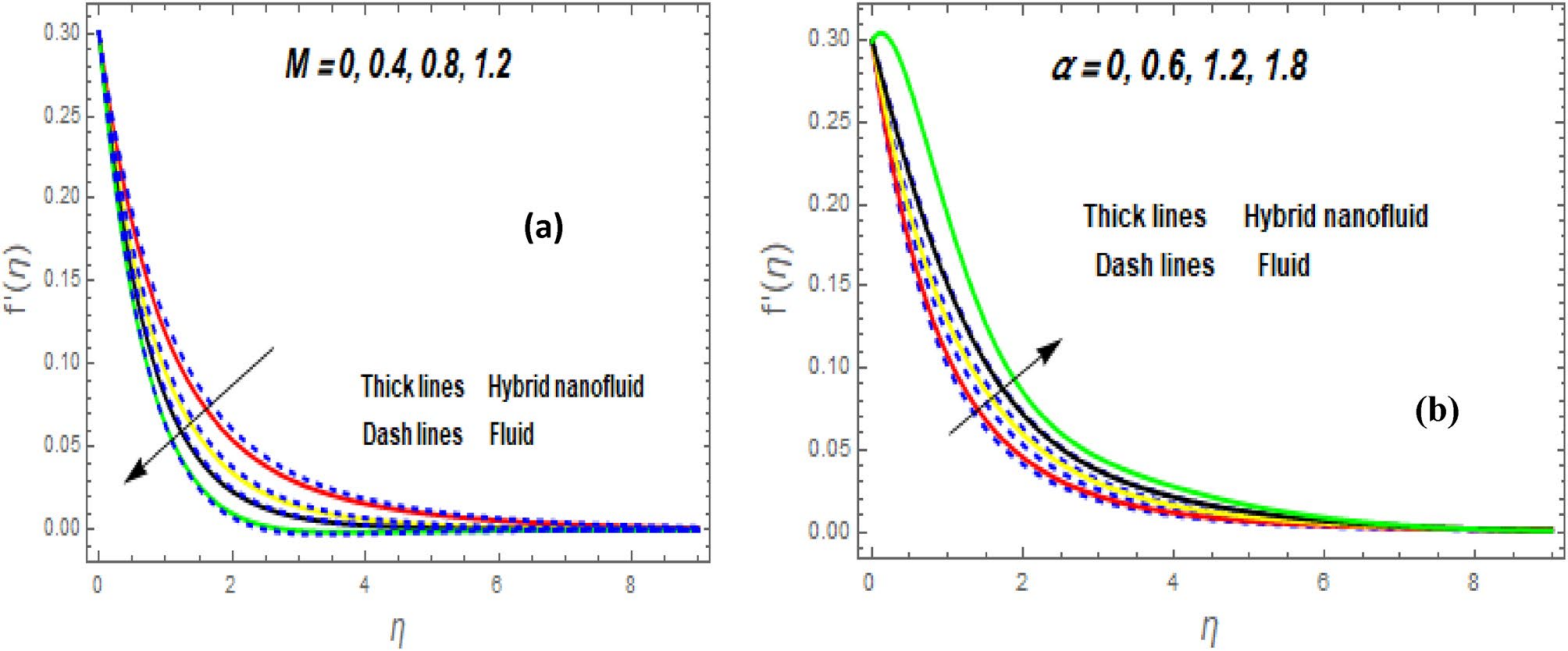
Impression of $f^{\prime}\left( \eta \right)$ (**a** and **b**) for *M* and $\alpha .$

**Figure 4:**
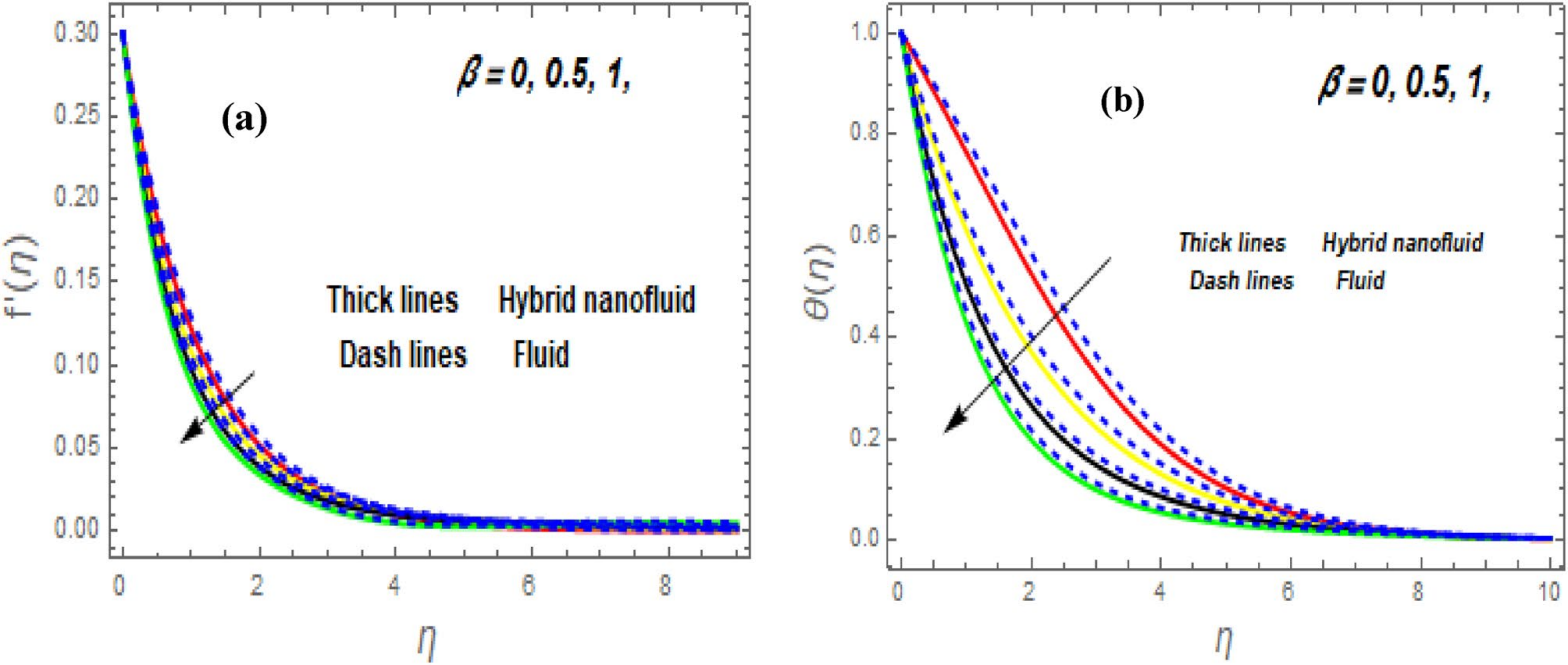
Impression of $f^{\prime}\left( \eta \right)$ (**a**) and $\theta \left( \eta \right)$ (**b**) for $\beta .$

**Figure 5:**
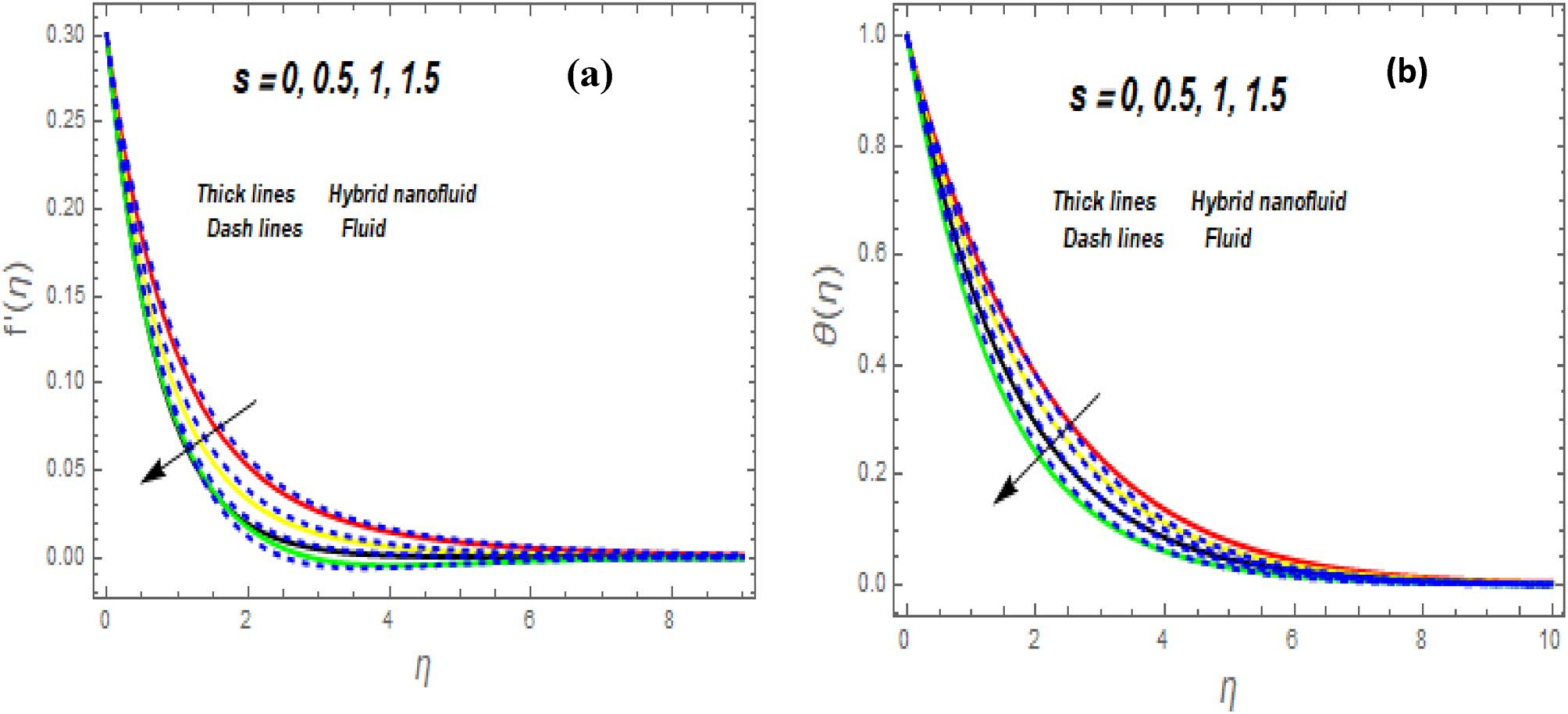
Impression of $f^{\prime}\left( \eta \right)$ (**a**) and $\theta \left( \eta \right)$ (**b**) for *s*.

**Figure 6:**
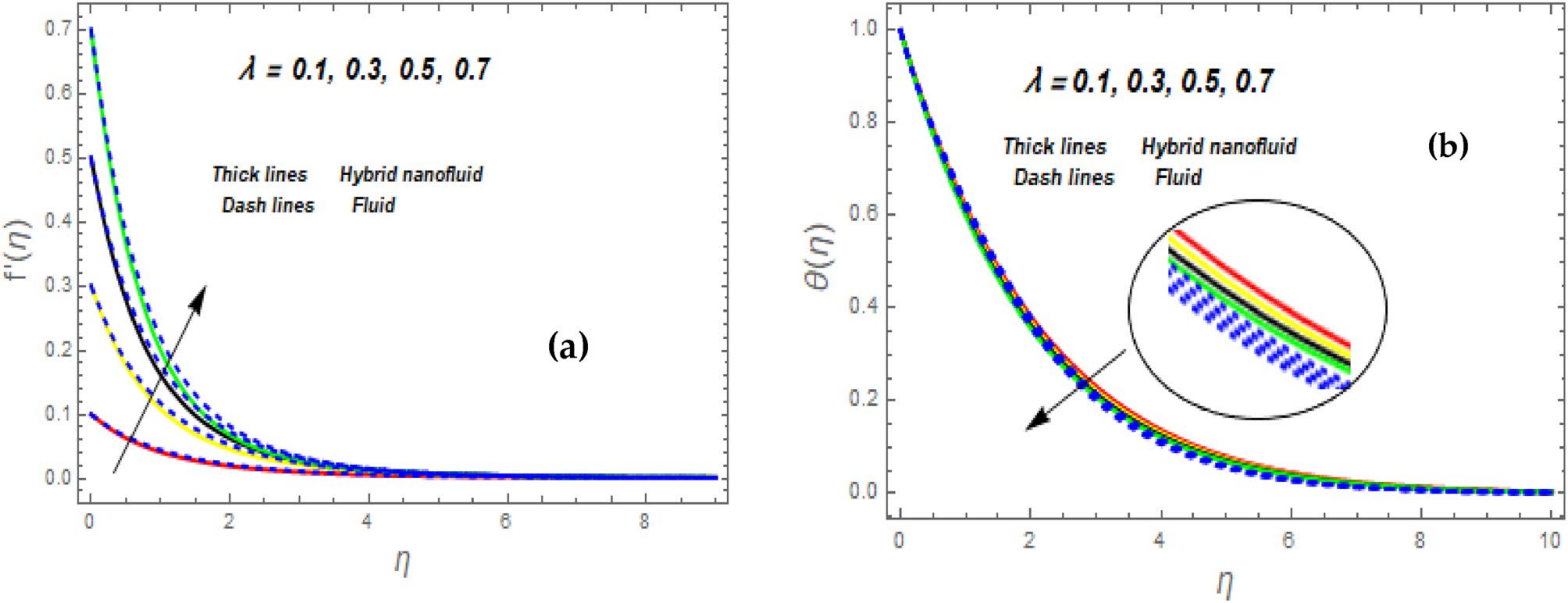
Impression of $f^{\prime}\left( \eta \right)$ (**a**) and $\theta \left( \eta \right)$ (**b**) for $\lambda .$

**Figure 7:**
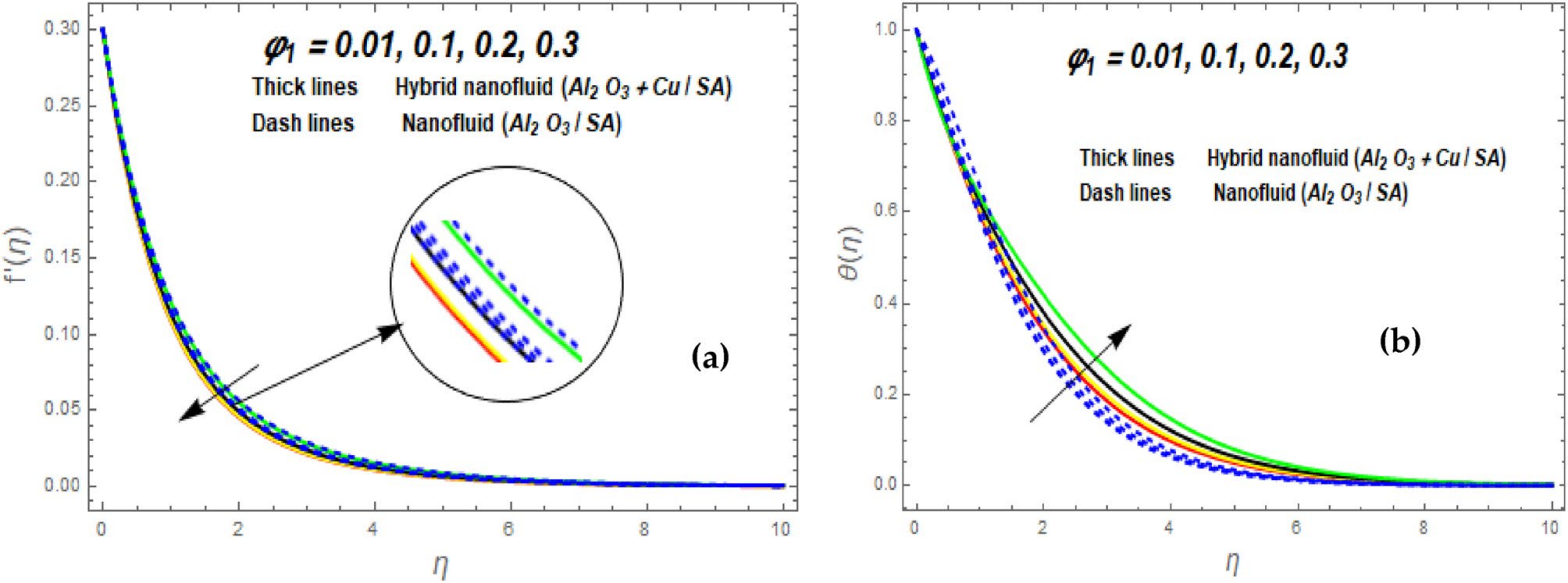
Impression of $f^{\prime}\left( \eta \right)$ (**a**) and $\theta \left( \eta \right)$ (**b**) for $\phi_{1} .$

**Figure 8:**
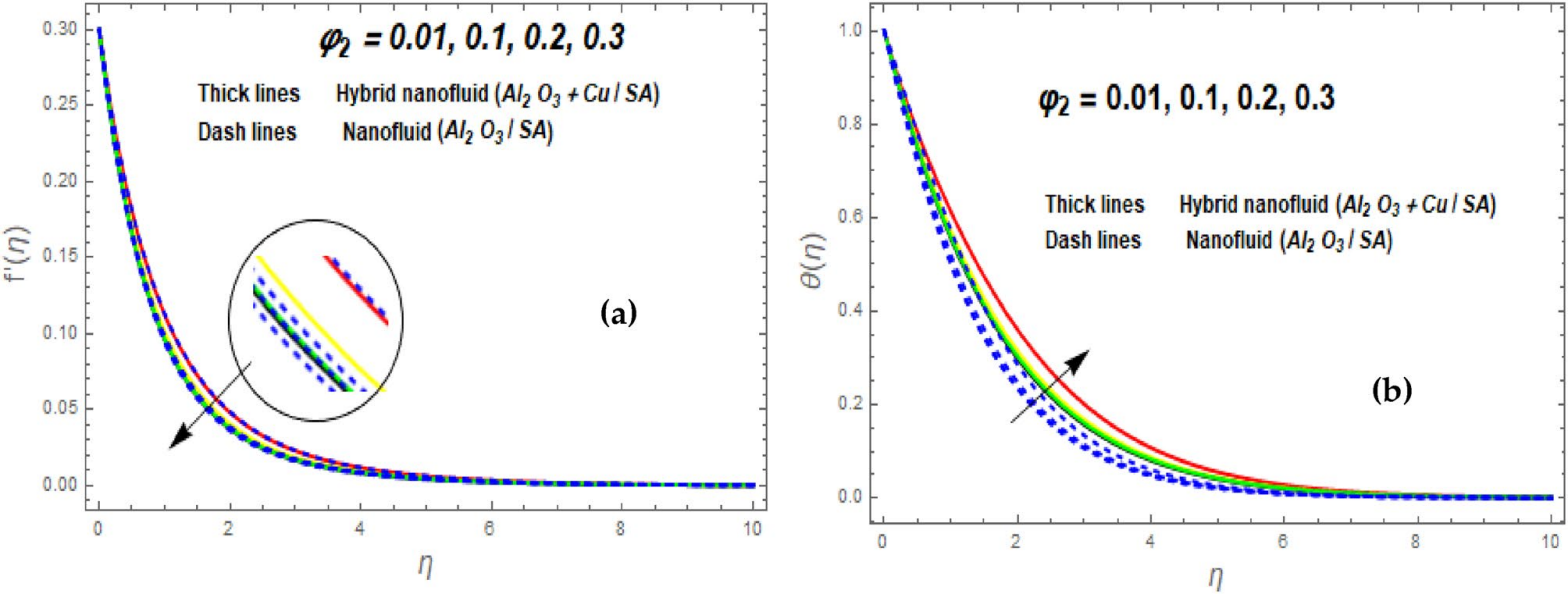
Impression of $f^{\prime}\left( \eta \right)$ (**a**) and $\theta \left( \eta \right)$ (**b**) for $\phi_{2} .$

**Figure 9:**
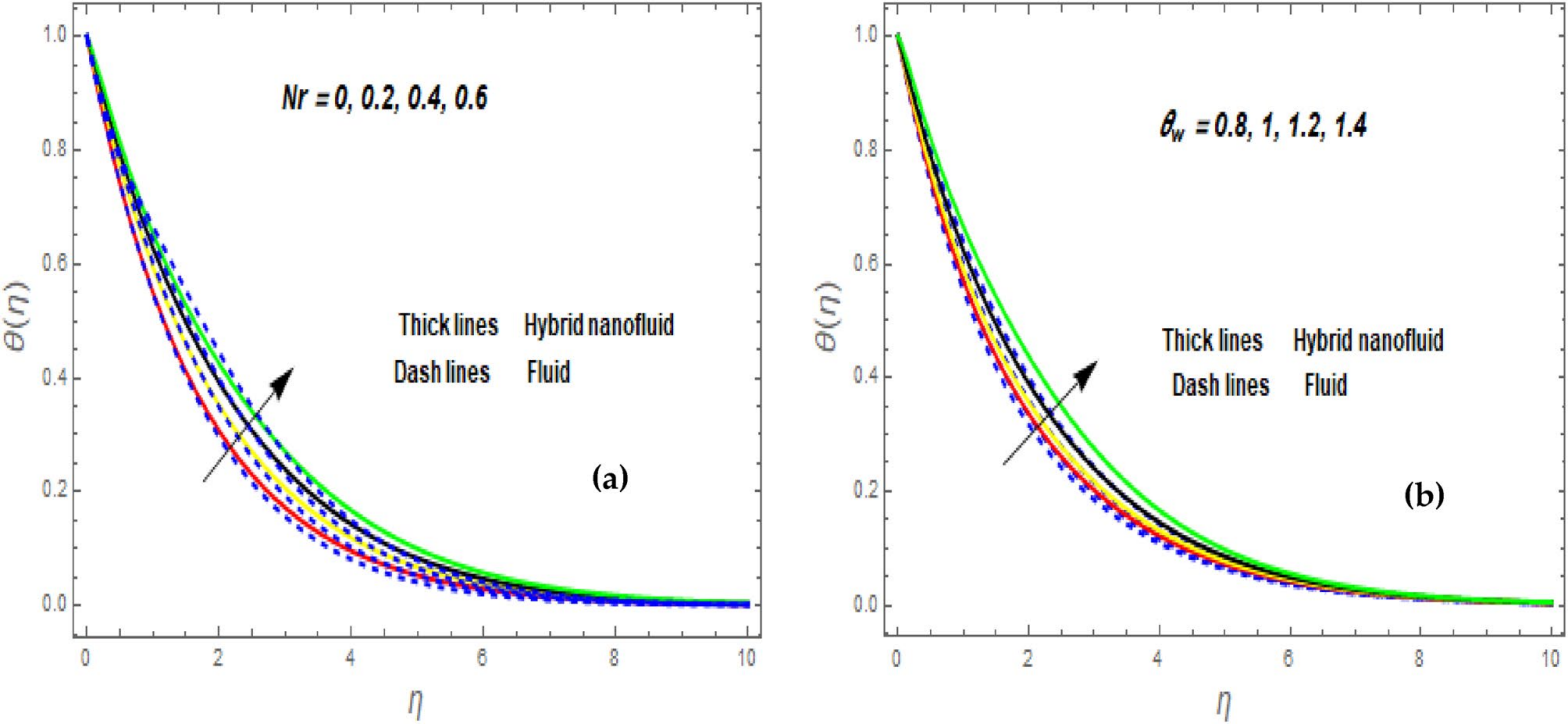
Impression of $\theta \left( \eta \right)$ (**a** and **b**) for *Nr* and $\theta_{w} .$

**Figure 10:**
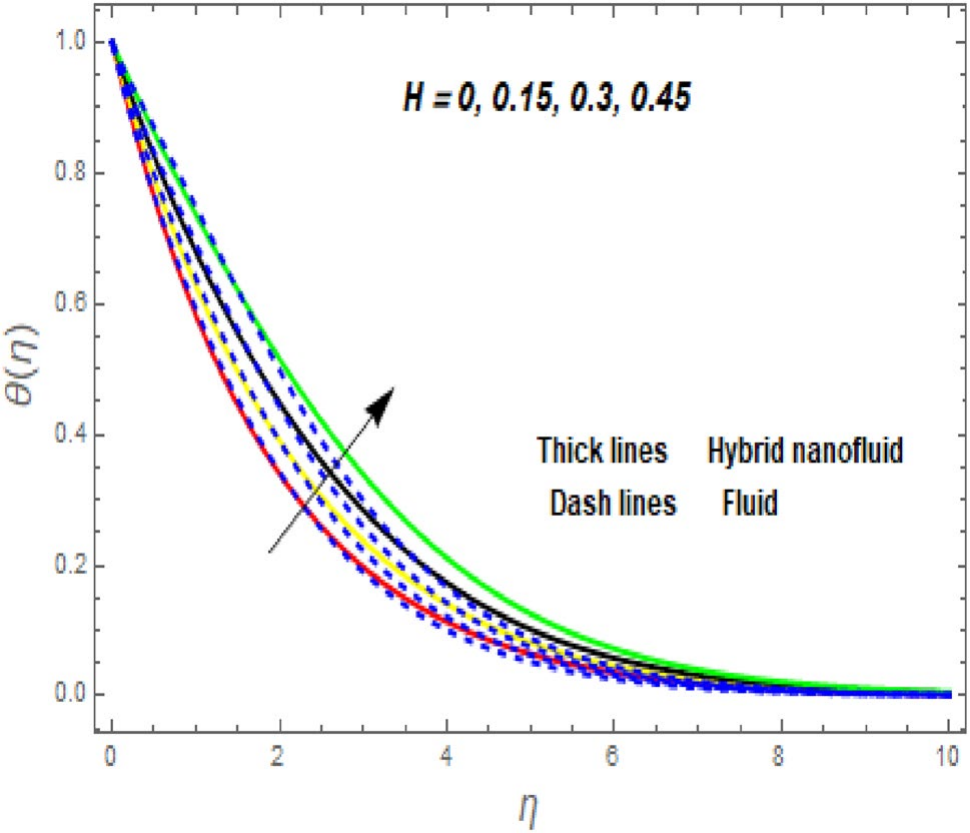
Impression of $\theta \left( \eta \right)$ for *H*.

Figure 3a illustrates how the magnetic (*M*) parameter controls the velocity field. For greater values of *M*, the flow rate dropped in both cases. Lorentz pressure is responsible for this phenomenon, which arises from the cooperation of electric and magnetic fields during an electrically conducted fluid flow. So, the fluid velocity in the BL is controlled by the generated Lorentz force. As a result, as *M* rises, the velocity of the fluid and hybrid nanofluids falls. The interaction of magnetic fields is significant in different technical and industrial applications such as crude oil extraction, geothermal systems, groundwater hydrology, and so on. Figure 3b illustrates the variation of the second-grade parameter ($\alpha$) on mobility. The technical meaning of it is the square of the shear stress divided by the first normal stress difference. It is found that $\alpha$ increasing generates a boost in the velocity of both liquid and nanofluid hybrids. This is because viscous forces and fluids' density decrease when alpha grows. Figures 4 and 5a,b demonstrate the impact of an unsteady parameter ($\beta$) and the suction parameter (s) on the flow and temperature fields. The respective temperature and profiles of velocity drop as $\beta$ and s rise. This demonstrates that with higher levels of $\beta$, the rate of cooling is substantially faster. This is because rising both and s decreases momentum and thermal BL. This is consistent with the concept that suction reduces the thickness of the outermost layer of fluid. Figure 6a,b shows the impression of stretching/shrinking parameters $\left( \lambda \right)$ on velocity and temperature distributions. When $\lambda$ increased, the velocity also upsurges while the temperature diminishes. Because the stretching parameters are set to higher levels, the temperature and thickness of the BL are reduced. Due to the exposure of the cooler to the ambient fluid, the BL thickness reduces with growing values of stretching parameters. Figure 7a,b shows the variation of nanoparticle volume fraction $\left( {\phi_{1} } \right)$ on the velocity and temperature distributions. When $\phi_{1}$ has raised, the velocity declines while the temperature boosts up. The variability of volume fractional of nanoparticles $\left( {\phi_{2} } \right)$ on velocity and temperature gradients is exposed in Fig. 8a,b. When $\phi_{2}$ progress, speed drops while temperature upsurges. The main reason for the decay in the velocity is that as the values of volume fractional of nanoparticle growths, the resistive force also increases, reducing the fluid flow speed. Physically, the energy is discharged from the exponential sheet due to the resistive force of the nanoparticles. More energy is generated when more nanoparticles are added, causing the temperature to rise. Furthermore, the optimum temperature may be achieved because hybrid nanofluids have higher thermal conductivity than nanofluids. The characteristics of the radiation parameter *(Nr)* and the liquid temperature are seen in Fig. 9a,b. Electromagnetic radiation produced by the heat-driven movement of particles within materials is known as thermal radiation. Higher predictions of the *Nr* help in the unpredictable mobility of nanoparticles. Consequently, there have been greater collisions among particles and more energy released. As a consequence, the fluid heat rises. The thermal profile extends as the temperature ratio parameter $\left( {\theta_{w} } \right)$ grows, as shown in Fig. 9b. The findings illustrate that as *θw* increases, the temperature disparity $\left( {T_{w} - T_{0} } \right)$ increases and causes the fluid temperature to rise. Figure 10 pierces the heat generation parameter (H) impressions on the temperature field. The non-dimensional heat generation/absorption parameter is based on the heat produced or absorbed by the fluid. It is noticed that as the *H* > *0* grows, the temperature distribution improves. A physically higher thermal production value indicates that more heat is generated within the boundary layer, which produces higher temperatures in the field.

**Figure 11:**
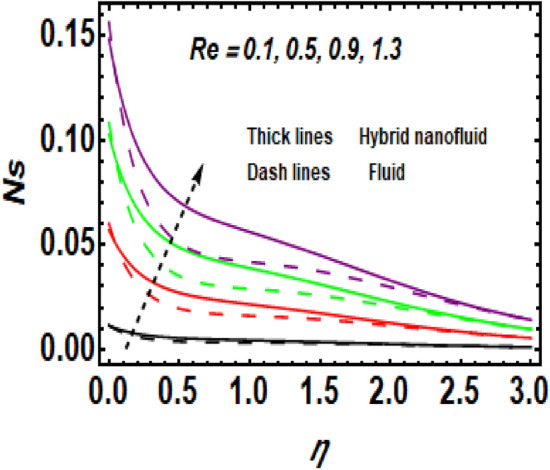
Impression of *Ns* for *Re*.

**Figure 12:**
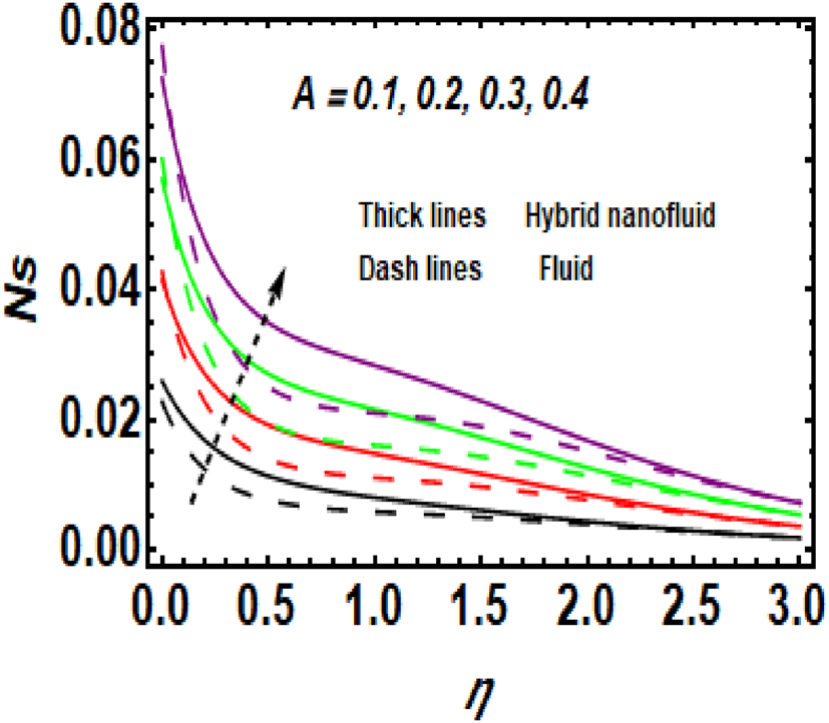
Impression of *Ns* for *A*.

**Figure 13:**
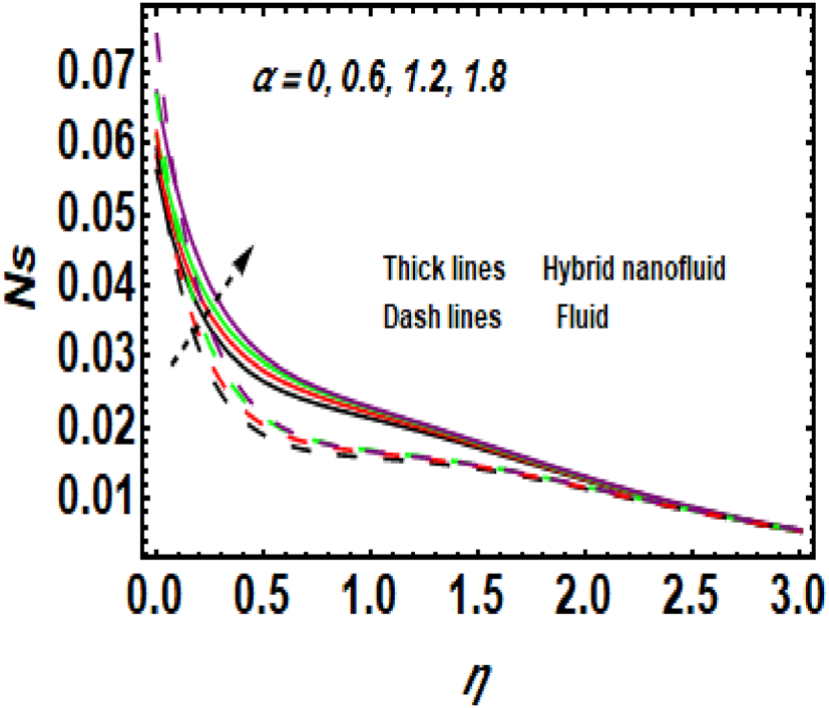
Impression of *Ns* for $\alpha .$

**Figure 14:**
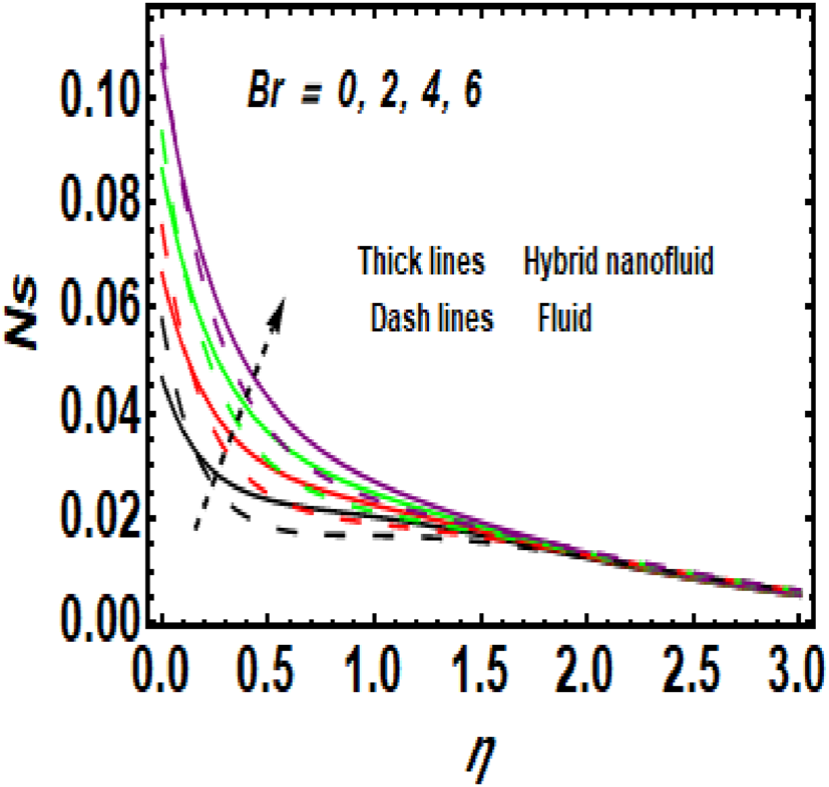
Impression of *Ns* for *Br*.

Figure 11 demonstrates the upshot of the Reynolds number (*Re*) on the entropy rate (*Ns*). The ratio of viscous forces to inertial forces in a fluid that experiences relative internal movement due to varying fluid velocities is described as the Reynolds number. A rise in Re causes the entropy rate to rise. Physically, since greater values of Re lead to larger inertial force and decay of the viscous force, the entropy rate hikes near the exponentially stretching surface. Figure 12 shows the temperature difference parameter (A) 's contribution to the entropy rate near the exponentially stretching sheet. When *A* is raised, then the entropy rate also boosts. Figure 13 influences the effect of $\alpha$ on *Ns*. It is seen that *Ns* upsurge due to the rise in $\alpha .$ Physical, Entropy formation is encouraged by stronger elastic effects. Figure 14 outlines the impact of the Brinkman number (Br) on the entropy rate (Ns). It is the proportion of heat transferred by molecular conduction to heat generated by viscous dissipation. The entropy rate is elevated via *Br*. Because *Br* and thermal conduction are inversely proportional. A larger value of Br causes low thermal conduction*,* consequently enhancing the entropy rate near the surface. Also, the hybrid nanoparticles enhance the entropy rate compared to a regular fluid. Hence, the quantity of heat energy is carried out.

**Figure 15:**
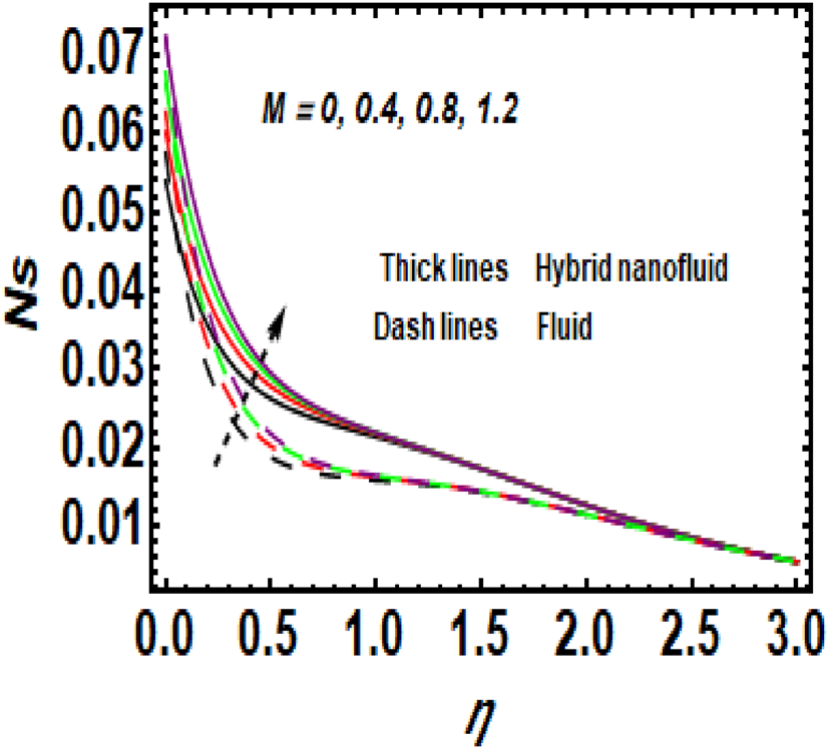
Impression of *Ns* for *M*.

Figure 15 the impression of varying values of *M* on *Ns* is shown. It was shown that the entropy rate improves as a result of *M*. The higher value of *M* leads to an upsurge in the resistance to the movement of liquid in the exponentially stretching sheet. Consequently, heat boosts and the entropy rate also rises.

**Figure 16:**
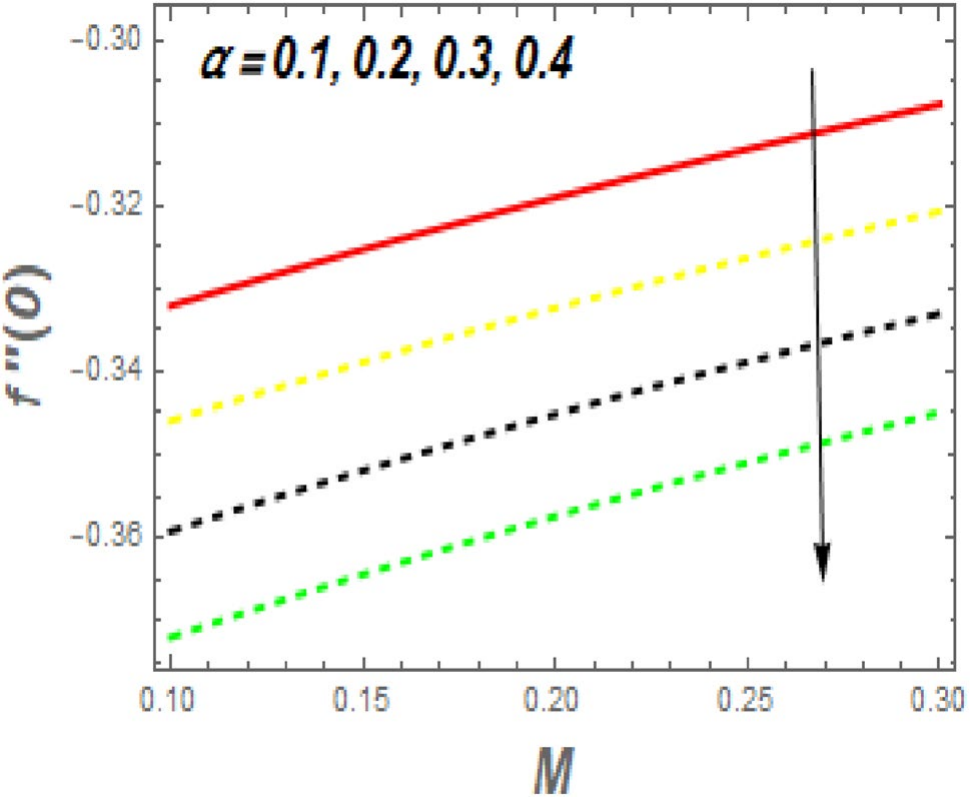
Impression of *M* and $\alpha$ on $C_{f} .$

**Figure 17:**
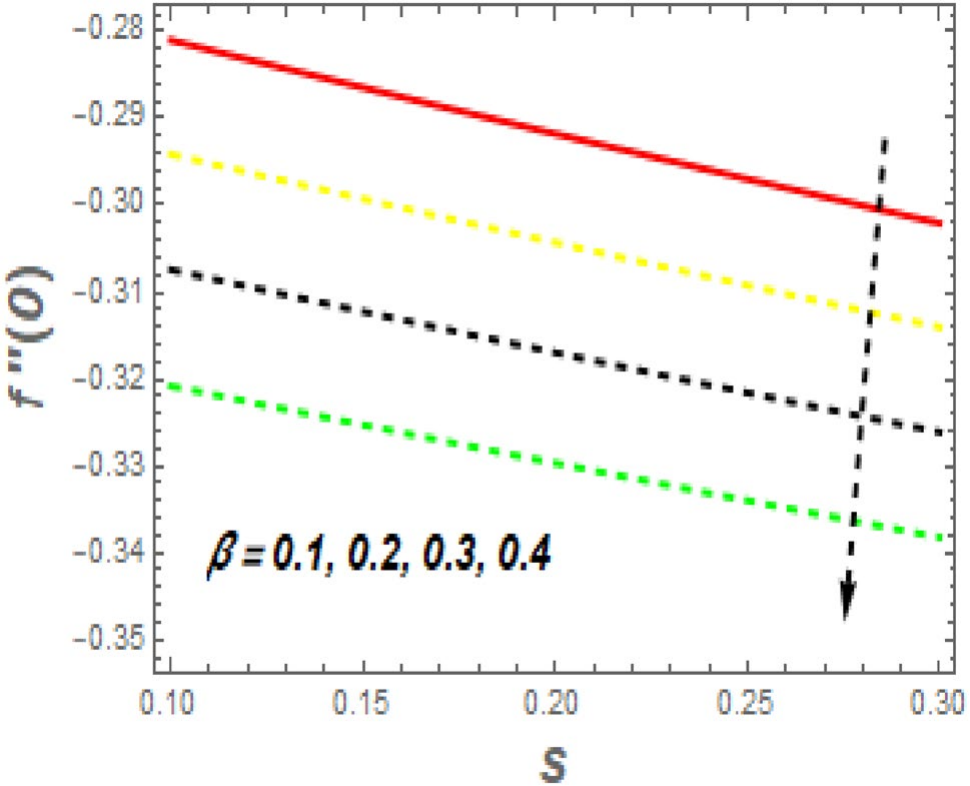
Impression of *s* and $\beta$ on $C_{f} .$

**Figure 18:**
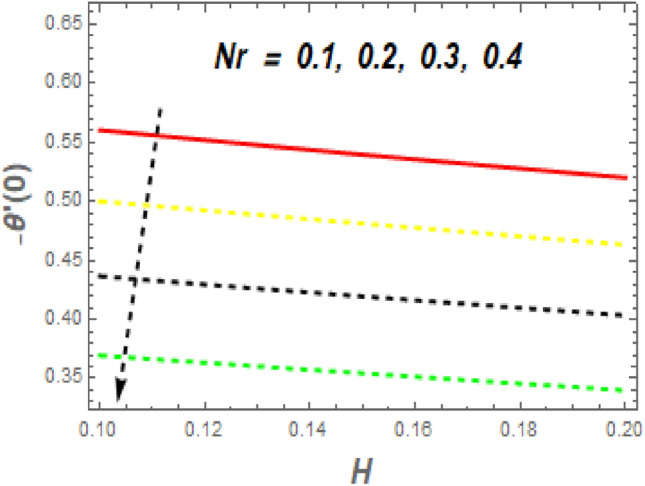
Impression of *Nr* and $H$ on $Nu.$

**Figure 19:**
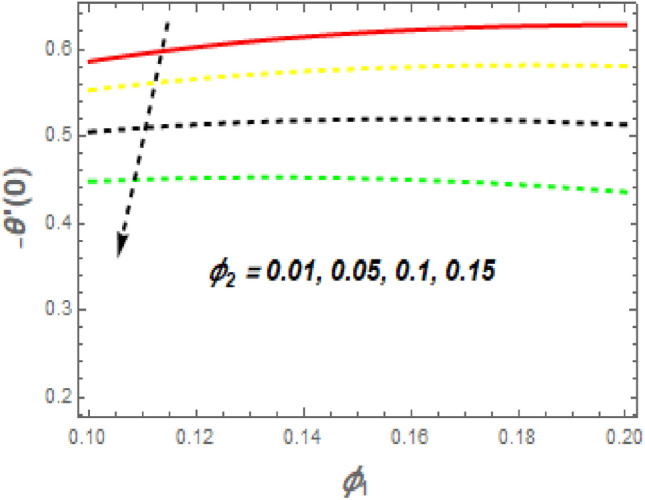
Impression of $\phi_{1}$ and $\phi_{2}$ on $Nu.$

As demonstrated in Fig. 16, f′′(0) increases as *M* increases, whereas it declines as $\alpha$ increases. Rising *M* generates substantial obstacles to fluid flow because of the Lorentz drag force, which decreases the velocity of the fluid and momentum BL width, raising velocity and, consequently, shear stress at the exponentially stretching sheet. The behavior of $f^{\prime\prime}\left( 0 \right)$ the unsteady parameter $\left( \beta \right)$ and suction/injection parameter (*s*) is revealed in Fig. 17. It can be detected the drag force declines with the rise in $\beta$ and *s*. Figure 18 impact of *Nr* and *H* on $Nu_{x} .$ It is observed that the $Nu_{x}$ reductions with an increase in *Nr* and *H*. The $Nu_{x}$ lower has $\phi_{1} \,\,{\text{and}}\,\,\phi_{2}$ increased, as shown in Fig. 19. Physically, the heat emitted from the exponential sheet when enhancing $\phi_{1} \,\,{\text{and}}\,\,\phi_{2} .$

### Fuzzy results and discussion

**Figure 20:**
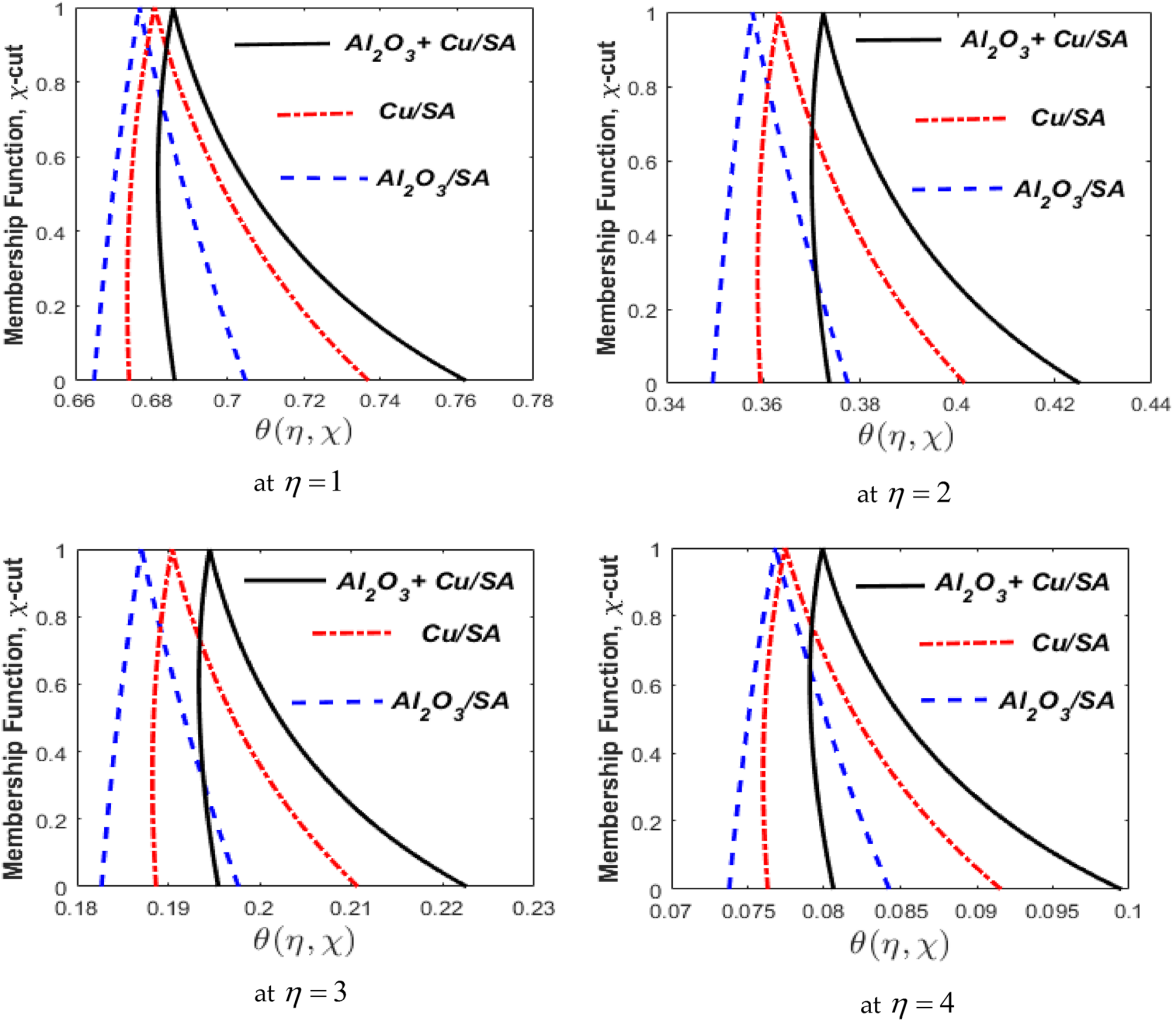
Comparison of nanofluids ${\text{Al}}_{{2}} {\text{O}}_{{3}} /{\text{SA,}}$ ${\text{Cu/SA}}$ and ${\text{Al}}_{{2}} {\text{O}}_{{3}} + {\text{Cu/SA}}$ hybrid nanofluids for $\omega = 0.5$ and different values of $\eta .$

Figure 20 portrays the calculated fuzzy temperature using volume fractions of $\phi_{1}$ and $\phi_{2}$ as the TFN [0%, 10%, 20%]. Four subdivisions for triangular MFs demonstrate the fuzzy temperatures for various thresholds of $\eta ,$ the following: 1, 2, 3, and 4. The MF of the fuzzy temperature profile $\zeta {\text{-cut}}\,\left( {0 \le \zeta {\text{-cut}} \le 1} \right),$ is displayed on the vertical axis, while the fuzzy temperature profile for different values of $\eta ,$ is displayed on the horizontal axis. The derived fuzzy temperatures are TFN, nevertheless not symmetrical, while the derived fuzzy volume fraction contains both TFN and symmetrical. These variations might be due to the nonlinearity of the governing FDE. It was also revealed that hybrid nanofluids had a wider width than nanofluids. As a result, the hybrid nanofluid is uncertain according to the TFN. On the other hand, Fig. 20 shows the comparison of nanofluids $Al_{2} O_{3} /SA$ $\left( {\phi_{1} } \right),$ $Cu/SA$ $\left( {\phi_{2} } \right),$ and $Al_{2} O_{3} + Cu/SA$ hybrid nanofluids through MF for numerous values of $\eta .$ In these figures, we evaluated three scenarios. Blue lines represent the situation in which $\phi_{1}$ is considered as TFN and $\phi_{2} = 0$. Whenever the $\phi_{2}$ is considered TFN and $\phi_{1} = 0$, it is represented by red lines. In the third case, black lines show the hybrid nanofluid that has both $\phi_{1}$ and $\phi_{2}$ non-zero. It has been determined that the hybrid nanofluid operates well since its thermal variation is greater than that of the other nanofluids. Physically, the combined thermal conductivities of $Al_{2} O_{3}$ and $Cu$ are incorporated in a nanofluid with a hybrid structure to offer maximal heat transmission. When discussing $Al_{2} O_{3} /SA$ and $Cu/SA$ nanofluids have $Al_{2} O_{3} /SA$ an enhanced heat transfer rate while $Al_{2} O_{3}$ having higher temperature conductivity than $Cu.$

## Conclusions

This study analyzed the unsteady MHD flow caused by exponentially stretching/shrinking surfaces in a second-grade hybrid $\left( {{\text{Al}}_{{2}} {\text{O}}_{{3}} + {\text{Cu/SA}}} \right)$ nanofluid. Alumina $\left( {{\text{Al}}_{{2}} {\text{O}}_{{3}} } \right)$ and copper (Cu) nanoparticles are mixed with sodium alginate to create a hybrid $\left( {{\text{Al}}_{{2}} {\text{O}}_{{3}} + {\text{Cu/SA}}} \right)$ nanofluid. Entropy generation, viscous dissipation, heat scores/sink, and non-linear thermal radiation are also considered. An analytical approach such as HAM is implemented for the outcome of the suggested problem. For validity, present results were found to be good when comparing existing results. The impacts of non-dimensional regulating parameters on velocity and temperature profiles for second-grade fluid and hybrid nanofluid are examined and discussed via graphs. Further, $\phi_{1} \,\,{\text{and}}\,\,\phi_{2}$ are said to be TFNs using the $\chi {\text{-cut}}$ technique. Comparison and uncertainty are studied through triangular fuzzy graphs. The foremost goals of this study are presented below:

- Due to strong Lorentz effects, the fluid velocity is dropped with the magnetic parameter, which is boosted with the second-grade fluid parameter. However, this retardation to the flow of hybrid nanofluid is higher than the retardation experienced by the flow of second-grade fluid.
- The fluid temperature is boosted while the velocity profile declines with the improvement of $\phi_{1} \,\,{\text{and}}\,\,\phi_{2} .$
- The fluid temperature boosts against higher $\theta_{w} ,$ *Nr*, and *H,* values, whereas the reverse holds for the unsteady parameter, suction parameter, and Prandtl number. However, the radiations emitted by the hybrid nanofluid are more potent than those emitted by a second-grade fluid.
- The fluid velocity grows with an upsurge in the stretching/shirking parameter, whereas the fluid temperature declines.
- The entropy profiles enhance as the second-grade fluid, Brinkman number, magnetic parameter, and Reynolds number rise.
- Heat transmission entropy effects dominate the exponentially stretching/shrinking sheet, whereas viscous dissipation and magnetic field entropy impacts dominate heat transmission entropy impacts in the distant region. Finally, we conclude that the entropy effects of heat transmission are the primary source of entropy production. The hybrid nanofluid shows maximum heat transfer in entropy production compared to the second-grade fluid.
- Skin friction coefficients are reduced with increased unsteady and second-grade parameters while growing with magnetic parameters.
- For higher values of *Nr*, *H*, $\phi_{1} \,\,{\text{and}}\,\,\phi_{2} ,$ the rate of heat transfer at the surface decreases.
- The variation among the lower and upper limits of fuzzy temperature profiles of hybrid nanofluids is highest for fuzzy analysis employing the triangle membership function. Hence, the degree of fuzziness is highest when compared to conventional nanofluids.
- Based on triangle fuzzy MF, ${\text{Al}}_{{2}} {\text{O}}_{{3}} + {\text{Cu/SA}}$ nanofluids that are hybrids are particularly able to enhance the temperature flow rate compared to ${\text{Al}}_{{2}} {\text{O}}_{{3}} /{\text{SA}}$ and ${\text{Cu/SA}}$ nanofluids in fuzzy heat transfer examinations. Moreover, when examined, the ${\text{Cu/SA}}$ nanofluid outperformed the nanofluid ${\text{Al}}_{{2}} {\text{O}}_{{3}} /{\text{SA}}$.

## Data Availability

All the data used and analyzed is available in the manuscript.

## Abbreviations

*u*,*v*: Velocity components
*x, y*: Cartesian coordinates
$f^{\prime}\left( \eta \right)$: Dimensionless Fluid velocity
*T*: Temperature of fluid
$\theta \left( \eta \right)$: Dimensionless Fluid Temperature
*T*_*w*_, *T*_∞_: Reference and ambient temperature
$$\eta$$: Similarity variable
$$\psi$$: Stream function
$\beta$: Shrinking/stretching rate parameter
*M*: Magnetic parameter
$\theta_{w}$: Temperature ratio parameter
*Ec*: Eckert number
$\alpha$: Second-grade fluid parameter
$$\Pr$$: Prandtl number
*H*: Heat source/sink parameter
$\mu_{f}$: Dynamic viscosity of the fluid
*k*: Thermal conductivity (unit: W/(m K))
$s_{2}$: Solid nanoparticles of Cu
$s_{1}$: Solid nanoparticles of Al_2_O_3_
$$\overline{\theta }(\eta ,\,\chi )$$: Fuzzy Temperature
$$f^{\prime}(\eta ,\,\chi )$$: Fuzzy velocity
FDE: Fuzzy differential equation
$$Nu_{x}$$: Nusselt number
$\chi$: Level or cut technique
$\rho_{hnf}$: Density of hybrid nanofluid
$\nu_{hnf}$: Kinematic viscosity of hybrid nanofluid
$\alpha_{hnf}$: Thermal diffusivity of hybrid nanofluid
$\left( {\rho C_{p} } \right)_{hnf}$: Heat capacity of hybrid nanofluid
$\delta_{hnf}$: Electrical conductivity of hybrid nanofluid
$$\delta_{f}$$: Electrical conductivity
$Nr$: Thermal radiation parameter
$\phi_{2}$: Volume fraction of copper
$\phi_{1}$: Volume fraction of alumina
$C_{fx}$: Skin-friction coefficient
${\text{Re}}_{x}$: Local Reynolds number
$\mu_{hnf}$: Dynamic viscosity of hybrid nanofluid
$A$: Dimensionless temperature gradient
$$N_{S}$$: Entropy generation
$$Br$$: Brinkman number
